# Inhibition of Autophagy Promotes the Elimination of Liver Cancer Stem Cells by CD133 Aptamer-Targeted Delivery of Doxorubicin

**DOI:** 10.3390/biom12111623

**Published:** 2022-11-03

**Authors:** Wang Yin, Cuong V. Pham, Tao Wang, Hadi Al Shamaileh, Rocky Chowdhury, Shweta Patel, Yong Li, Lingxue Kong, Yingchu Hou, Yimin Zhu, Sunrui Chen, Huo Xu, Lee Jia, Wei Duan, Dongxi Xiang

**Affiliations:** 1 IMPACT, Institute for Innovation in Physical and Mental Health and Clinical Translation, School of Medicine, Deakin University, Geelong, VIC 3216, Australia; 2 Telethon Kids Institute, University of Western Australia, Perth, WA 6009, Australia; 3 The College of Nursing and Health, Zhengzhou University, Zhengzhou 450001, China; 4 Institute for Immunology and Infectious Diseases, Murdoch University, Perth, WA 6150, Australia; 5 Cancer Care Centre, St George Hospital, Kogarah, and St George and Sutherland Clinical School, University of New South Wales Kensington, Kogarah, NSW 2217, Australia; 6 Institute for Frontier Materials, Deakin University, Waurn Ponds, VIC 3216, Australia; 7 Laboratory of Tumor Molecular and Cellular Biology College of Life Sciences, Shaanxi Normal University, 620 West Chang’an Avenue, Xi’an 710119, China; 8 CAS Key Laboratory of Nano-Bio Interface, Suzhou Institute of Nano-Tech and Nano–Bionics, Chinese Academy of Sciences, Suzhou 215123, China; 9 Shanghai OneTar Biomedicine, Shanghai 201203, China; 10 College of Materials and Chemical Engineering, Minjiang University, Fuzhou 350108, China; 11 State Key Laboratory of Oncogenes and Related Genes, Shanghai 200127, China; 12 Department of Biliary-Pancreatic Surgery, Renji Hospital Affiliated to Shanghai Jiao Tong University School of Medicine, Shanghai 200127, China; 13 Shanghai Key Laboratory of Biliary Tract Disease Research, Shanghai 200092, China

**Keywords:** hepatocellular carcinoma, cancer stem cells, doxorubicin, autophagy, CD133, aptamer

## Abstract

Doxorubicin is the most frequently used chemotherapeutic agent for the treatment of hepatocellular carcinoma. However, one major obstacle to the effective management of liver cancer is the drug resistance derived from the cancer stem cells. Herein, we employed a CD133 aptamer for targeted delivery of doxorubicin into liver cancer stem cells to overcome chemoresistance. Furthermore, we explored the efficacy of autophagy inhibition to sensitize liver cancer stem cells to the treatment of CD133 aptamer-doxorubicin conjugates based on the previous observation that doxorubicin contributes to the survival of liver cancer stem cells by activating autophagy. The kinetics and thermodynamics of aptamer-doxorubicin binding, autophagy induction, cell apoptosis, and self-renewal of liver cancer stem cells were studied using isothermal titration calorimetry, Western blot analysis, annexin V assay, and tumorsphere formation assay. The aptamer-cell binding andintracellular accumulation of doxorubicin were quantified via flow cytometry. CD133 aptamer-guided delivery of doxorubicin resulted in a higher doxorubicin concentration in the liver cancer stem cells. The combinatorial treatment strategy of CD133 aptamer-doxorubicin conjugates and an autophagy inhibitor led to an over 10-fold higher elimination of liver cancer stem cells than that of free doxorubicin in vitro. Future exploration of cancer stem cell-targeted delivery of doxorubicin in conjunction with autophagy inhibition in vivo may well lead to improved outcomes in the treatment of hepatocellular carcinoma.

## 1. Introduction

Multidrug resistance is one of the main causes of chemotherapy failure in hepatocellular carcinoma (HCC). Accumulating evidence has shown that a small subset of liver cancer cells, termed liver tumor-initiating cells or liver cancer stem cells, are responsible for the initiation, propagation, metastasis, and treatment resistance in liver cancer [1]. Differing from bulk cancer cells, this liver cancer stem cell subpopulation displays resistance to traditional chemotherapeutic drugs via overexpression of the ATP-binding cassette transporters (i.e., ABCB1, ABCG2, and ABCC1), which serve as pumps to actively expel small molecular drugs [2].

Aptamers, also known as chemical antibodies, are short single-stranded oligonucleotides that can bind to their targets with high affinity and specificity. Compared with traditional antibodies, aptamers possess several advantages, such as low production cost, minimal batch-to-batch variation, lack of immunogenicity, prolonged shelf life, and ease of incorporating chemical modifications for enhanced binding properties [3]. During the past three decades, various aptamer-based drug delivery systems for either oligonucleotide therapeutics (i.e., siRNA, antimir, antisense oligonucleotides) or chemotherapeutics have been reported. In the current work, we endeavor to improve the targeting of liver cancer stem cells of doxorubicin (DOX) via conjugating DOX to an aptamer against CD133, a confirmed liver cancer stem cell marker with elevated expression in liver cancer cells [4].

Recent studies have revealed the pro-survival roles of autophagy in liver cancer stem cells in the hypoxic and nutrient-deprived tumor microenvironment [5]. The inhibition of autophagy led to the potentiation of the tumoricidal effects of chemotherapy, implying that inhibition of autophagy may sensitize liver cancer stem cells to DOX.

In this study, the anti-hepatocellular carcinoma stem cell effect of the combined treatment of CD133 aptamer-DOX and autophagy inhibitors was explored. Our results indicate that while CD133 aptamer-DOX conjugate alone enables effective targeting and penetration of liver cancer stem cells, the concomitant inhibition of autophagy inhibition can further potentiate the capacity of CD133 aptamer-DOX in eliminating liver cancer stem cells.

## 2. Materials and Methods

### 2.1. Cell Culture

Huh7 cell line (human HCC, Japanese Collection of Research Bioresources) was kindly provided by Dr. Liang Qiao (University of Sydney, Australia). The PLC/PRF/5 (human HCC, ATCC CRL-8024) cell line was purchased from ATCC. These cells were cultured in DMEM medium supplemented with 10% fetal bovine serum and 1 × Glutamax (Life Technologies, Gaithersburg, MD, USA) in a humidified atmosphere containing 5% CO_2_ at 37 °C.

### 2.2. Generation and Characterization of the CD133 Aptamer-Doxorubicin Conjugates (CD133 Aptamer-DOX)

The development of CD133 aptamer-DOX, and the determination of the molar ratio of aptamers to DOX, DOX-loading efficiency, and stability of CD133 aptamer-DOX were performed according to our previous publication [6] with modifications described in the Supplementary Materials.

### 2.3. Isothermal Titration Calorimetry (ITC)

ITC experimental parameters were set up as described in our previous publication [7], with details described in the Supplementary Materials.

### 2.4. Determination of Binding Affinity

The equilibrium dissociation constant (*K_D_*) of CD133 aptamer to CD133-positive and CD133-negative cells was determined using flow cytometry described in our previous publication [8], with details presented in the Supplementary Materials.

### 2.5. Annexin V Assay

The 7-AAD/Annexin V assay was used to determine the early- and late-stage apoptosis of cells. The cells were trypsinized and stained with annexin V (BioLegend, San Diego, CA, USA; #640918, 1:20 dilution) and 7-AAD (BioLegend, San Diego, CA, USA; #420404, 1:20 dilution) for 15 min at room temperature in the dark to determine cell apoptosis. Apoptosis was evaluated by 7-AAD/Annexin V assay using flow cytometry. The cells in both the early and late stages of apoptosis were considered apoptotic cells.

### 2.6. In Vitro Tumorsphere Formation Assay

The tumorsphere assay was conducted according to our previously published protocol [6] with modifications described in the Supplementary Materials.

### 2.7. Statistical Analysis

All statistical analyses were performed using GraphPad Prism 8.0 (San Diego, CA, USA). An unpaired *t*-test was used for comparisons between two experimental groups, and one-way analysis of variance (ANOVA) was used for comparisons of more than two groups. All analyses were two-tailed. Data normality was tested by the Kolmogorov–Smirnov test, and parametrical statistical tests were only carried out if normality was confirmed. The homogeneity of variance was tested by Bartlett’s test. The Dunnett or the Tukey post hoc tests were conducted to compare every mean to a control mean or with every other mean only if the F value in ANOVA achieved *p* < 0.05, and there was no significant variance in homogeneity. Otherwise, the data were converted to logarithms for further analysis. Unless otherwise indicated, all results were averaged from biological triplicates, and values are reported as means ± SD. *p* < 0.05 was considered statistically significant.

## 3. Results

### 3.1. Development and Characterization of a CD133 Aptamer-DOX Conjugate

**Figure 1:**
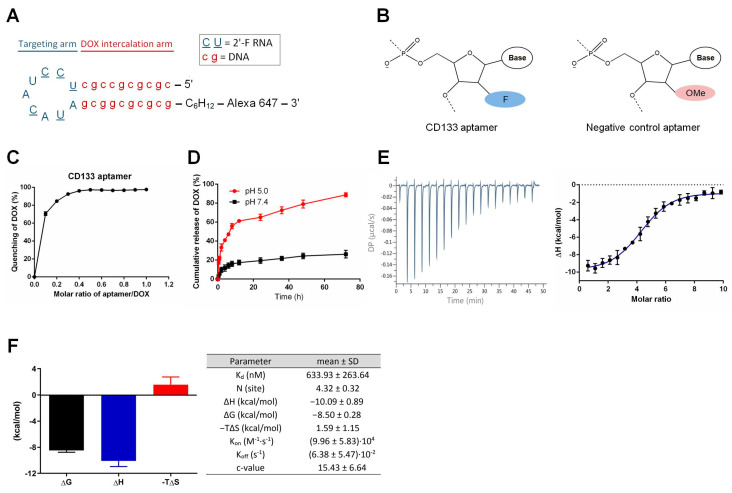
Schematic illustration of the hybrid RNA-DNA CD133 aptamer and characterization of the CD133 aptamer-doxorubicin conjugates (CD133-DOX). (**A**) The predicted secondary structure of the CD133 aptamer. A 10-bp DNA GC stem was engineered to replace the original RNA stem in the RNA aptamers, along with a 2′-fluoropyrimidine modification of all pyrimidines, 5′-methyl-dC in the stem, and an Alexa 647 fluorophore conjugation to the 3′-end of the CD133 aptamer. A hexylamine cap of the CD133 aptamer was added to the 3′-end for enhanced nuclease resistance. (**B**) The negative control aptamers are aptamers of the same sequence as the CD133 aptamer but with a 2′-O-methyl (OMe) modification instead of a 2′-fluoropyrimidine (F) modification at the pyrimidines (C and U), which changes the three-dimensional structure of the aptamer and thus abolishes the binding of the control aptamers to CD133. (**C**) In vitro pH-dependent DOX release from CD133-DOX at a pH of 5.0 and 7.4. (**D**) The intercalation of DOX into the CD133 aptamer. The fluorescence quenching of DOX (10 µM) after incubation with different aptamer concentrations (1, 2, 3, 4, 5, 6, 7, 8, 9, and 10 µM, respectively) for 30 min. (**E**) Isothermal titration calorimetry (ITC) of interaction between CD133 aptamer and DOX. Left, representative raw calorimetric data for titration of CD133 aptamer (2 µM) with serial injections of DOX solution (100 µM) (left). Right, binding isotherms resulting from the integration of the raw calorimetric data. (**F**) Left, the thermodynamic signature for titration by stepwise injection of DOX into CD133 aptamers. Right, thermodynamic parameters for DOX binding to CD133 aptamers obtained by ITC at 25 °C. Data shown are means ± SD, (*n* = 3).

DOX is known to preferentially bind to double-stranded 5′-GC-3′ or 5′-CG-3′ sequences of DNA [9]. Previous studies in our laboratory showed that the loop of the CD133 aptamer is the main determinant for target binding [4]. Thus, the original RNA stem in the aptamers was replaced by a DNA segment consisting of 10 repeated GC pairs for DOX loading while maintaining the binding competence of the aptamer (Figure 1A). 2′-O-methyl (OMe) modification has been shown to alter the three-dimensional structure of an RNA aptamer and thus abolish its binding [10]. Therefore, an aptamer with the same sequence as the CD133 aptamer but with a 2′-OMe modification instead of a 2′-fluoropyrimidine (F) modification at the pyrimidines (C and U) was adopted as a negative control.

To determine the optimal molar ratio for optimal preparation of the DOX-aptamer conjugation, we prepared the aptamer-DOX conjugates with different molar ratios of aptamers to DOX. We measured DOX fluorescence using a plate reader. As shown in Figure 1C, the fluorescence of DOX was progressively quenched with the increasing molar ratios of aptamers to DOX. The fluorescence quenching reached the plateau (>90% of quenching) at the molar ratio of aptamer: DOX of 0.27, indicating that approximately four DOX molecules were conjugated with one CD133 aptamer molecule. The efficiency of DOX loading onto aptamers was determined to be 96.2 ± 2.1%.

As a targeted therapy, the CD133 aptamer-DOX complex must remain stable in the systemic circulation. On the other hand, the DOX should be released swiftly from the aptamer after being endocytosed by the CD133-positive liver cancer cells. To investigate the pH-dependent loading of DOX to the CD133 aptamer, we used PBS (pH 7.4) to mimic the pH in the blood and acidified PBS (pH 5.0) to simulate the acidic environment in the lysosomes. As shown in Figure 1D, less than 15% of DOX was released from the CD133 aptamer-DOX after incubation with PBS (pH 7.4) for 8 h. In contrast, DOX was rapidly released from the CD133 aptamer-DOX after only 2 h incubation at a pH of 5.0. Specifically, ~89% of intercalated DOX was released from the aptamer after 72 h at pH 5.0, compared to a 26.2% release after 72 h at the pH of 7.4. The pH-dependent release of DOX is critically essential for CD133 aptamer-DOX. It could potentially minimize the systemic exposure of DOX to sensitive organs in the circulation system, particularly to reduce cardiotoxicity, but allows rapid drug release upon entry of CD133 aptamer-DOX conjugates into the CD133-positive cancer cells.

To study the kinetic and thermodynamic properties of the aptamer-DOX binding, we employed isothermal titration calorimetry (ITC) for further characterization of the binding. As shown in Figure 1E, the binding of DOX to CD133 aptamers resulted in a negative peak of differential power, indicating that the aptamer-DOX interaction is exothermic. This finding was further confirmed by the thermodynamic profile of the aptamer-DOX binding, as well as the kinetic and thermodynamic parameters shown in Figure 1F. The validity of the ITC study is routinely confirmed by the Wiseman c value, in which an optimal curve fitting results in a c between 10–500 [11]. In our experimental system, the desirable c values ranging from 10.37 to 22.95 were achieved (Figure 1F).

The equilibrium dissociation constant (*K_D_*), dictated by the changes in Gibbs free energy (Δ*G*), measures the tendency of a large complex to dissociate into its components. A low *K_D_* value indicates a high affinity of the ligand for the target. The *K_D_* for CD133 aptamer binding to DOX was 634 nM (the right panel of Figure 1E), which is considered medium affinity [12]. Such a medium *K_D_* value is desirable for maintaining the stability of the CD133 aptamer-DOX complex in systemic circulation while promoting effective dissociation of DOX from the CD133 aptamer after endocytosis.

The stoichiometry parameter *N* represents the number of ligands that bind to the target. In our ITC study, the *N* value was measured to be 4.32, indicating that approximately four DOX molecules bound to one CD133 aptamer (Figure 1F). This finding is consistent with the data from Figure 1C, which shows that the molar ratio of DOX vs. aptamer was approximately 4 in the aptamer-DOX conjugates.

Enthalpy changes (Δ*H*) reflect the change in the number and strength of the non-covalent bond from the free state to the bound state and can be measured by ITC. Entropy change (Δ*S*) is the change in entropy from desolvation and conformational changes upon binding and can be calculated as Δ*S* = (Δ*H* − Δ*G*)/*T*. In our study, a negative Δ*H* and Δ*S* value were determined, indicating ample involvement of hydrogen bonding and unfavorable conformational changes in this reaction, respectively (Figure 1F). Moreover, the fact that the enthalpy changes (Δ*H*) were higher than the change of entropy (−*T*Δ*S*) indicates that the CD133 aptamer-DOX binding is mainly driven by enthalpy changes.

Next, we determined the association rate constant (*K_on_*) and the dissociation rate constant (*K_off_*) of the binding between the CD133 aptamer and DOX with the aid of AFFINImeter software [13]. Compared with the published *K_on_* and *K_off_* values obtained from aptamer-ligand interaction in the literature [14], our aptamer-DOX interaction exhibited a lower *K_on_* value and higher *K_off_* value (Figure 1F). Such values indicate that once bound, DOX can be readily released from the aptamer-DOX conjugates in the desired environment. Indeed, such prediction is in accordance with our experimentally determined dissociation of DOX from CD133 aptamer-DOX (Figure 1D).

### 3.2. The CD133 Aptamer Specifically Binds to CD133-Positive Human Liver Cancer Cells

**Figure 2:**
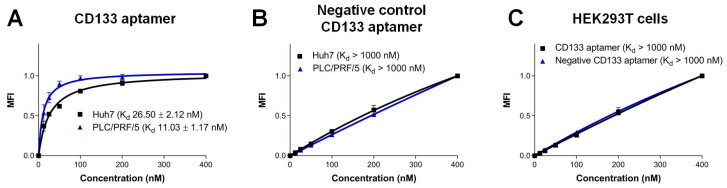
Characterization of the specificity of the CD133 aptamer. Alexa Fluor 647-labeled CD133 aptamers were incubated with indicated human cells and analyzed by flow cytometry. The median fluorescence intensity (MFI) was plotted against varying concentrations of CD133 aptamer (1–400 nM). (**A**) Binding of CD133 aptamer to CD133-positive Huh7 and PLC/PRF/5 cells. (**B**) Binding of the negative control CD133 aptamers to Huh7 and PLC/PRF/5 cells. (**C**) Binding of CD133 aptamers to CD133-negative HEK293T cells. Data shown are means ± SD, (*n* = 3).

The CD133 aptamer used in this project was previously developed in our laboratory [4]. Here, we investigated the interaction between CD133 aptamer and CD133-positive expression cell lines using flow cytometry. Using an anti-CD133 antibody, we found that 35.4% of Huh7 cells and 15.5% of PLC/PRF/5 cells expressed CD133 (Supplementary Figure S1). The Huh7 and PLC/PRF/5 cells used in the current study may represent HCC cells from different pathological backgrounds. The difference in the origin of these two cell lines would help to mimic the heterogeneity of the primary cells, although not completely [15]. For example, our previous studies show that these two cell lines have different surface expression levels of drug efflux pumps (MDR1 and ABCG2), as well as different levels of cancer stem cell surface markers (EpCAM and CD133) [16]. Furthermore, the PLC/PRF/5 cell line was established from a patient with hepatitis B virus (HBV) infection, which is among the main risk factors for HCC [17]. The HBV-encoded X antigen (HBx) has been shown to promote stemness and chemoresistance via activating the PI3K/AKT signal pathway [18,19,20]. In contrast, Huh7 was established from a patient without HBV infection [21,22], representing the cohort of HCC patients with a very different etiology from those infected with HBV. Indeed, the overall survival rate of HCC patients without HBV is significantly better than that of HBV-HCC [23]. The apparent *K_D_* for the CD133 aptamer towards the liver cancer Huh7 and PLC/PRF/5 cell lines was determined as ~27 nM and ~11 nM, respectively (Figure 2). In contrast, there was a minimum binding of the CD133 aptamer to the CD133-negative HEK293T cell line, evidently forming a *K_D_* of >1000 nM. The specificity of the interaction was further demonstrated by the lack of binding to target cells by a negative control CD133 aptamer (*K_D_* > 1000 nM, Figure 2), which has an identical nucleotide sequence but with an altered three-dimensional structure due to a different side-chain chemical modification at 2′-pyrimidines (Figure 1B). Furthermore, upon binding to target cells, the CD133 aptamers were efficiently internalized into CD133-positive liver cancer cells via receptor-mediated endocytosis (Supplementary Figure S2).

### 3.3. Enhanced Delivery of DOX into Liver Cancer Stem Cells via CD133 Aptamer

To ensure the conjugation of DOX molecules does not compromise the binding capacity of the original aptamer sequence, it is critical to evaluate if CD133 aptamer-DOX conjugates can successfully deliver DOX into the CD133-expressing liver cancer cells. To this end, we used flow cytometry to determine the intracellular accumulation of DOX in the liver cancer stem cells, which can be phenotypically defined by the double immunostaining of the cell surface CD133 and epithelial cell adhesion molecule (EpCAM) [24].

**Figure 3:**
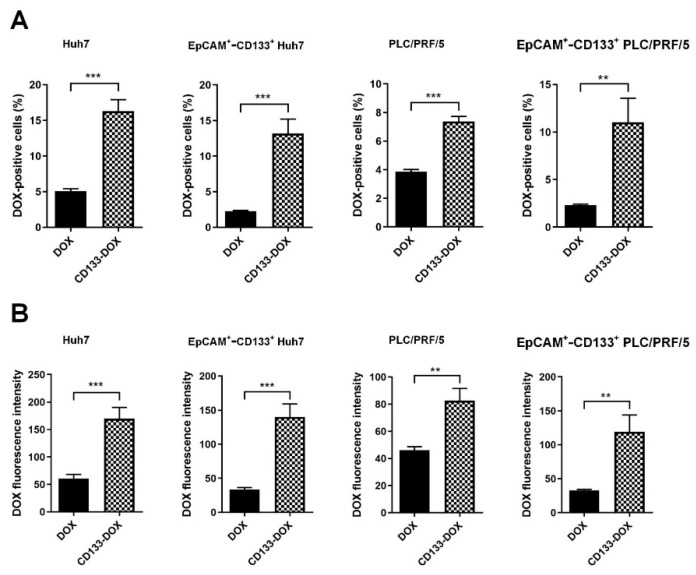
DOX accumulation in the bulk and EpCAM^+^-CD133^+^ population of the liver cancer Huh7 cells and PLC/PRF/5 cells. After incubation with free DOX or CD133 aptamer-DOX conjugates (200 nM for Huh7 and 100 nM for PLC/PRF/5) for 24 h, the intracellular accumulation of DOX was analyzed via flow cytometry. (**A**) The percentage of DOX-positive cells, and (**B**) the corresponding median intracellular DOX fluorescence intensity. Data shown are means ± SD, *n* = 3. ** *p* < 0.01; *** *p* < 0.001, compared to free DOX treatment.

CD133 aptamer-mediated delivery of DOX was found to accomplish a higher intracellular DOX than free DOX, evident from the percentage of DOX-positive cells and cognate DOX fluorescence intensity (Figure 3 and Supplementary Figures S3 and S4). Specifically, there was at least an ~180% or 80% increase in intracellular DOX in Huh7 and PLC/PRF/5, respectively. Importantly, CD133 aptamer-mediated delivery of DOX resulted in at least 300% or 260% increases in intracellular DOX in the EpCAM^+^-CD133^+^ subpopulation of Huh7 and PLC/PRF/5 cells, respectively.

Next, we sought to verify the ability of the CD133 aptamers to deliver DOX into bulk liver cancer cells and liver cancer sphere-forming cells, which are in vitro surrogates of liver cancer stem cells [25], using alternative experimental approaches. To this end, semi-quantitative assessment via confocal microscopy (Supplementary Figure S5) and quantitative high-performance liquid chromatography (HPLC) assays were employed (Supplementary Figure S6). Consistent with the findings from fluorescence-based studies (Figure 3), the intracellular concentration of DOX delivery by CD133 aptamer-DOX conjugates in the bulk and sphere-forming liver cancer cells was found to be several folds higher than that in cells treated with an equivalent concentration of free DOX (*p* < 0.01) (Supplementary Figures S5 and S6).

Thus, the CD133 aptamer enhances the delivery and intracellular retention of DOX to the bulk of liver cancer cells in general and the liver cancer stem cells in particular.

### 3.4. Autophagy Inhibition Augmented Apoptosis Elicited by DOX

DOX has been reported to induce apoptosis in bulk cancer cells in a time- and dose-dependent manner [26]. We next investigated if the elevated intracellular concentration of DOX achieved via the treatment of CD133 aptamer-DOX conjugates can translate into elevated chemotherapeutic efficacy. Furthermore, accumulating preclinical data have revealed the capacity of autophagy inhibition to reverse DOX resistance in various cancer types, suggesting the inhibition of autophagy as one of the promising therapeutic strategies [5]. Therefore, we wished to explore if autophagy inhibition by a pharmacological inhibitor or RNAi could lead to a synergized promotion of apoptosis in combination with DOX. For this purpose, a 7-AAD and Annexin V assay was employed to gauge the extent of apoptosis.

**Figure 4:**
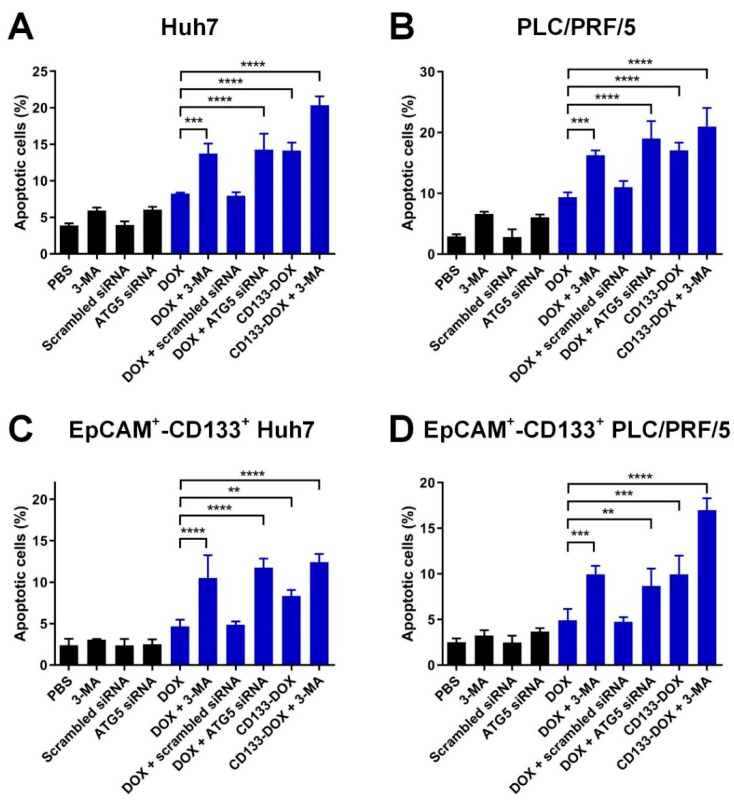
The effect of the inhibition of autophagy combined with the treatment of CD133 aptamer-DOX conjugates on DOX-induced apoptosis. The cells were treated with autophagy inhibitor 3-MA (2 mM) or ATG5 siRNA (200 nM) followed by the addition of either free DOX or an equivalent dose of CD133 aptamer-DOX conjugates (200 nM for Huh7 and 100 nM for PLC/PRF/5) for 24 h. The total percentage of apoptotic cells was defined by the combination of the percentage of 7-AAD^-^/Annexin V^+^ and 7-AAD^+^/Annexin V^+^ cells and are shown for the (**A**) bulk Huh7 cells, (**B**) bulk PLC/PFR/5 cells, (**C**) EpCAM^+^-CD133^+^ Hun7 cells, as well as (**D**) EpCAM^+^-CD133^+^PLC/PRF/5 cells. Data shown are means ± SD, *n* = 3. ** *p* < 0.01; *** *p* < 0.001; **** *p* < 0.0001; compared with that in the DOX-only treatment.

First, we used RNAi to inhibit autophagy by transfecting cells with ATG5 siRNA and achieved a >75% reduction in autophagy activity as measured by the formation of the LC3-II protein (Supplementary Figure S8). Of note, the inhibition of autophagy itself did not increase DOX accumulation in either the EpCAM^+^-CD133^+^ population or bulk of the HCC cells (Supplementary Figure S9). As illustrated in Figure 4 and Supplementary Figures S10 and S11, the treatment with CD133 aptamer-DOX conjugates led to an approximately 70% and 100% increase in the percentage of apoptotic cells in the EpCAM^+^-CD133^+^ population of Huh7 and PLC/PRF/5 cells, respectively, compared with those treated with free DOX. Surprisingly, the combined treatment with DOX plus autophagy inhibition using the pharmacological inhibitor 3-MA or siRNA against ATG5 further increased apoptosis by 66% in the bulk HCC cells and 150% in the EpCAM^+^-CD133^+^ population of the HCC cells, respectively.

Next, an alternative experimental strategy, i.e., the TUNEL assay, was employed to further confirm the efficacy of the combinatorial treatment of CD133 aptamer-DOX conjugates and 3-MA in eliciting apoptosis in tumorsphere-forming cells of Huh7 and PLC/PRF/5. Consistent with the flow cytometry-based assay data (Figure 4), the microscopy-based TUNEL assays revealed that CD133 aptamer-mediated delivery of DOX resulted in approximately 180% more apoptosis in HCC tumorsphere-forming cells than those treated by free DOX. Moreover, when combined with 3-MA, CD133 aptamer-DOX conjugates achieved a further increase in apoptosis in the liver cancer sphere-forming cells by at least ~140% and ~300% compared with that by free DOX alone or CD133 aptamer-DOX alone, respectively (Supplementary Figure S12).

Therefore, the delivery of DOX via the CD133 aptamer as CD133 aptamer-DOX conjugates has enhanced the potency of DOX in inducing apoptosis in both the bulk and cancer stem cell subpopulation of HCC cells, whereas the concomitant inhibition of autophagy further enhanced the therapeutic efficacy of DOX in these cells.

### 3.5. Enhanced Elimination of Liver Cancer Stem Cells via CD133-Targeted Delivery of DOX and Inhibition of Autophagy

Having established that the combination of the increased and persistent intracellular dose of DOX delivered by a CD133 aptamer and inhibition of autophagy resulted in enhanced apoptosis of liver cancer stem cells, we proceeded to evaluate if such a favorable pharmacological and pharmacodynamic effect could translate into the therapeutic outcome of eliminating liver cancer stem cells. To this end, we employed an in vitro tumorsphere formation assay to assess the therapeutic impact of our treatment on a key functional feature of cancer stem cells, namely the ability of self-renewal [27].

**Figure 5:**
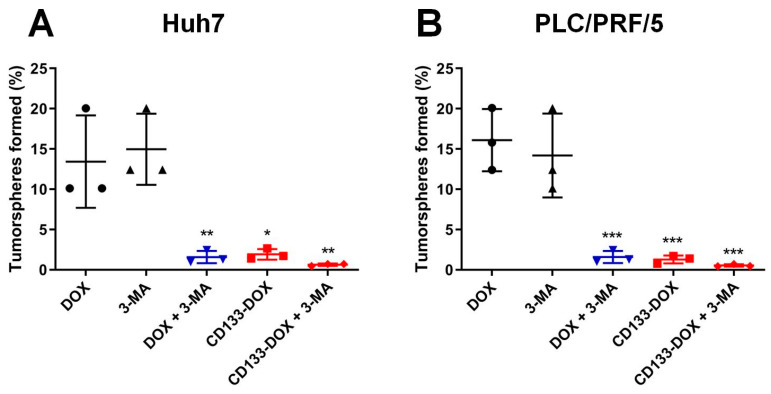
The percentage of tumorsphere formation determined 5–7 days after the treatment. The cells were treated separately with 3-MA (2 mM), DOX (200 nM for Huh7 and 100 nM for PLC/PRF/5), CD133 aptamer-DOX conjugates (equivalent to 200 nM or 100 nM DOX for Huh7 or PLC/PRF/5, respectively), 3-MA plus DOX or 3-MA plus CD133-DOX for 48 h. Cells were then plated onto ultra-low attachment 96 plates in stem cell culture media. The self-renewal capacity of cells in various treatment groups was analyzed using an in vitro limiting dilution assay. The frequency of tumorsphere-forming cells for (**A**) Huh7 and (**B**) PLC/PRF/5 under various treatments is shown. Data presented are means ± SD, *n* = 3. * *p* < 0.05; ** *p* < 0.01; *** *p* < 0.001, compared with DOX-only treatment.

As shown in Figure 5, in the saline control group, tumorspheres formed from both the Huh7 and PLC/PRF/5 cell lines had a tumorsphere frequency of 100% (Supplementary Table S1). In contrast, cells treated with free DOX or 3-MA alone showed a moderate decrease in the frequency of tumorsphere formation than those treated with the vehicle control (saline). Compared with the treatment with free DOX, the delivery of DOX in the form of CD133 aptamer-DOX conjugates resulted in an approximately 6-fold enhanced reduction in tumorsphere formation in the bulk Huh7 and PLC/PRF/5 cells, respectively (Figure 5).

Since autophagy is involved in the maintenance of stem cells [28], it is also vital to explore if autophagy inhibition could augment the capacity of DOX to eliminate liver cancer stem cells. To this end, the inhibition of autophagy was found to significantly potentiate the capacity of free DOX to eradicate liver cancer stem cells by nearly 7-fold in Huh7 and 9-fold in PLC/PRF/5, respectively (Figure 5). Moreover, the combinatorial treatment using autophagy inhibitor 3-MA and CD133 aptamer-DOX conjugates ensured a further reduction in tumorsphere formation frequency by 2.2-fold in Huh7 or 1.3-fold in PLC/PRF/5 cells, compared to those treated with CD133 aptamer-DOX conjugate alone (Supplementary Table S1). Taken together, these results indicate that DOX delivered as a CD133 aptamer-DOX conjugate can effectively impair the self-renewal of liver cancer stem cells, while a further boost in the efficacy of DOX in eliminating liver cancer stem cells can be accomplished by the inhibition of autophagy.

## 4. Discussion

Current strategies in treating liver cancer patients largely focus on eliminating the bulk cancer cells, which often fail as the cancer stem cells can survive chemotherapy, and tumor recurrence inevitably follows. The transmembrane protein CD133 has been identified as a marker for liver cancer stem cells [29]. A series of anti-CD133 antibodies have been developed for targeted therapy, including the anti-human CD133 monoclonal antibody 6B6 [30] and the CD133 antibody-toxin conjugates ^C178A^BC-CD133Mab, dCD133KDEL, dEpCAMCD133KDEL, and CD133-paclitaxel, which have exhibited high efficacy to eliminate tumors in vitro and in vivo [31]. Among these drugs, CD133KDEL entered Phase I clinical trials in 2016 (NCT02845414). However, the limited penetration of monoclonal antibodies into solid tumors restricts their anti-tumor efficacy due to their large size [31]. Our previous work has shown that the aptamer against the epithelial cell adhesion molecule (EpCAM) exhibited tumor penetration capacity in xenograft tumor tissue that was at least four-fold of that of the EpCAM antibody due to the smaller size of aptamers [8]. In the current study, we delivered chemotherapeutic agents into the CD133^+^ HCC cells using the CD133 aptamer, aiming at improving the effective intracellular concentration of chemotherapy agents in HCC cells.

**Figure 6:**
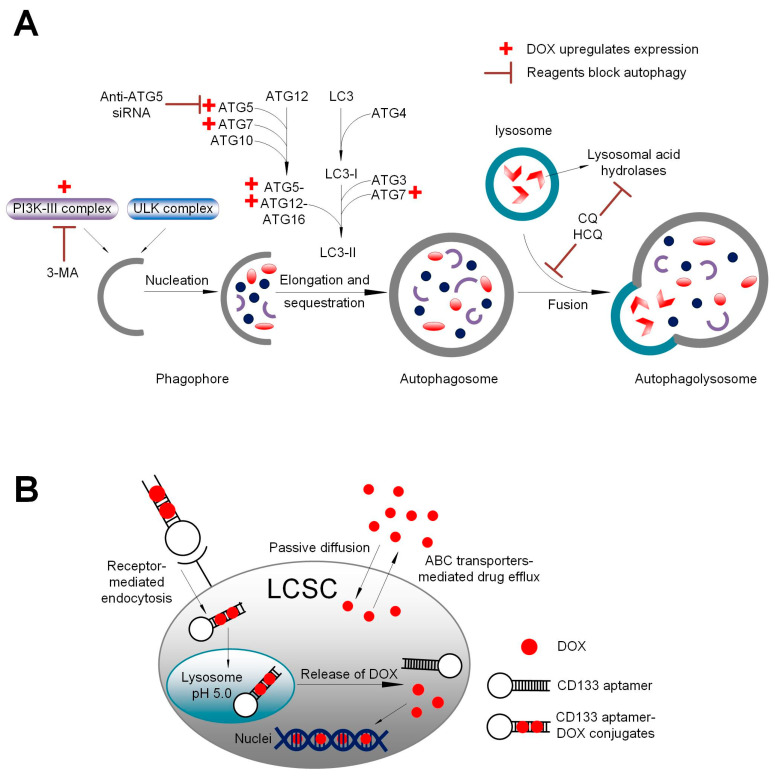
A schematic summary. (**A**) Schematic illustration of the impact of DOX, autophagy inhibitor 3-methyladenine (3-MA), and downregulation of autophagy-related 5 (ATG5) on autophagy. Class III phosphoinositide 3-kinase (PI3K-III) complex and ATG5 are critical components in autophagosome formation. DOX upregulates the formation of PI3K-III complex and ATG5, thus facilitating autophagosome formation. In contrast, 3-MA inhibits the formation of PI3K-III complex, while anti-ATG5 siRNA downregulates ATG5 expression. (**B**) Schematic summary of aptamer-guided targeted delivery of DOX into liver cancer stem cells. Upon binding to the CD133-positive cancer cells, the aptamer-conjugated DOX is efficiently internalized via receptor-mediated endocytosis, thus bypassing the drug efflux mediated by ABC transporters on the plasma membrane. Following endocytosis, DOX is released from the CD133 aptamer from endolysosomes. The free DOX is able to gain entry into the nuclei and induce apoptosis via binding to the DNA and poisoning topoisomerase II. LCSC: liver cancer stem cells; ABC transporter: ATP-binding cassette transporter.

There has been debate on whether CD133^+^ cancer cells are cancer stem cells, as CD133-negative glioma and colon cancer cells are tumorigenic in immunocompromised mice [32]. Studies by Kemper et al. [33] have shed light on this by demonstrating that AC133, a glycosylated form of CD133 epitope rather than the CD133 protein itself, is the marker for cancer stem cells. Indeed, experiments investigating CD133 in HCC have detected the AC133 epitope (CD133/1) rather than the CD133 protein [34]. The CD133 aptamer used in this study has been demonstrated to target the AC133 epitope [4], leading to an increased concentration of DOX in the liver cancer stem cells via the CD133 aptamer-mediated delivery of DOX, compared with that of free DOX (Figure 3 and Figure 6).

Our laboratory has reported on an engineered EpCAM aptamer with the same stem structure of 10 GC pairs as the CD133 aptamer used in the current investigation for DOX intercalation [6]. Interestingly, here we found that the CD133 aptamers are able to load twice as much DOX as the EpCAM aptamers do (Figure 1) [6]. One possible explanation is that this different DOX-loading efficacy could be attributed to their different 3′-ends: EpCAM aptamer has an inverted deoxythymidine (idT) cap while the CD133 aptamer has a hexylamine cap, which may lead to different three-dimensional structures in the stem of these two aptamers. The potential interaction between the loop and stem of the aptamer may contribute to the difference between the enhanced DOX loading to the stem between the CD133 aptamer-DOX in this study and the less efficient DOX loading to the stem of the EpCAM aptamer, as previously reported [6].

In this study, we show that the addition of the autophagy inhibitor 3-MA dramatically augmented the capacity of Dox or CD133 aptamer-DOX to eliminate liver cancer stem cells via compromising DOX-induced autophagy activation (Figure 4 and Figure 5, Supplementary Figure S8). Notably, the treatment of 3-MA alone seems to have a minimum effect on the autophagic activity of HCC cells. Wu et al. have reported that 3-MA plays a dual role in autophagic activity. On one hand, 3-MA can not only promote autophagy activity when treated under nutrient-rich conditions with a prolonged treatment period (up to 9 h), but also suppress starvation-induced autophagy [35]. In the current study, the HCC cells were incubated with 3-MA for two days, which is much longer than the treatment period reported by Wu et al. [35]. Therefore, the longer treatment period in the current study might explain the different effects on the autophagic activity as determined by the expression of LC3-II.

In oncologic clinics, increased autophagy response has been found in advanced HCC, correlating with malignant progression and poor prognosis [36]. Notably, several studies revealed the protective role of autophagy in liver cancer stem cells. For example, Song et al. have reported the involvement of autophagy in the maintenance of CD133^+^ liver cancer stem cells in hypoxic and nutrient-deprived conditions and the autophagic inhibitor chloroquine (CQ) increased cell apoptosis and decreased clonogenic capacity of CD133^+^ liver cancer stem cells [5]. In the clinical management of liver cancer, DOX-based transarterial chemoembolization, instead of systemic administration, is the current standard treatment for intermediate-stage HCC. The findings from this study have shown the combinatorial treatment of CD133 aptamer-DOX and autophagy inhibition leads to enhanced elimination of the bulk HCC cells and more importantly, the cancer stem cell population of HCC cells compared to free DOX. Thus, upon verification in future in vivo studies, the CD133 aptamer-mediated delivery of DOX combined with autophagy inhibitor hydroxychloroquine may constitute a novel approach to transarterial chemoembolization to improve the therapeutic outcome of patients with intermediate-stage HCC.

## Data Availability

The data presented in this study are available in the main text, figures, tables and supplementary material.

## Supplementary Materials

The following supporting information can be downloaded at: https://www.mdpi.com/article/10.3390/biom12111623/s1, Figure S1: The expression of cancer stem cell markers EpCAM and CD133 on Huh7 and PLC/PRF/5 cells; Figure S2: CD133 aptamer is endocytosed via receptor-mediated endocytosis; Figure S3: Representative flow cytometric diagrams showing DOX accumulation in the bulk population of (A) Huh7 or (B) PLC/PRF/5 cells; Figure S4: Representative flow cytometric diagrams showing DOX accumulation in the EpCAM^+^-CD133^+^ population of (A) Huh7 or (B) PLC/PRF/5 cells; Figure S5: Cellular uptake of DOX and CD133 aptamer-DOX in (A) Huh7, (B) PLC/PRF/5, and (C) HEK293T cells; Figure S6: In vitro tumorsphere assay; Figure S7: Evaluation of the impact of DOX and 3-MA on autophagy; Figure S8: ATG5 knockdown induced inhibition of autophagic activity; Figure S9: Flow cytometric measurement of DOX accumulation in the bulk and EpCAM^+^-CD133^+^ population of Huh7 and PLC/PRF/5 cells; Figure S10: The contour diagram of 7-AAD/Annexin V flow cytometry; Figure S11: The contour diagram of 7-AAD/Annexin V flow cytometry of the EpCAM^+^-CD133^+^ population; Figure S12. Induction of apoptosis in the sphere-forming liver cancer cells; Table S1. Effect of autophagy inhibition on the capability of DOX or CD133 aptamer-DOX to eliminate cancer stem cells in vitro. References [37,38,39,40,41] are cited in the supplementary materials

## Abbreviations

EpCAM: epithelial cell adhesion molecule; 3-MA, 3-methyladenine; HCC, hepatocellular carcinoma.
