# Supplementary material for: Inhibition of Autophagy Promotes the Elimination of Liver Cancer Stem Cells by CD133 Aptamer-Targeted Delivery of Doxorubicin
Source: Biomolecules. 2022 Nov 3;12(11):1623. doi: 10.3390/biom12111623 (PMC9687680; doi:10.3390/biom12111623)
Supplement: Supplementary file 1 [file biomolecules-12-01623-s001.zip › biomolecules-1920751-supplementary.pdf]

Supplementary Materials

Title: Inhibition of autophagy promotes the elimination of liver cancer stem cells by CD133 aptamer-targeted delivery of doxorubicin

Authors: Wang Yin[1#Δ], Cuong V. Pham[1#], Tao Wang[2, 3], Hadi Al Shamaileh[4], Rocky Chowdhury[1], Shweta Patel[1], Yong Li[5], Lingxue Kong[6], Yingchu Hou[7], Yimin Zhu[8], Sunrui Chen[9], Huo Xu[10], Lee Jia[10], Wei Duan[1*], Dongxi Xiang[11,12,13*],

Affiliations:

[1]Deakin University, School of Medicine, IMPACT, Institute for Innovation in Physical and Mental Health and Clinical Translation, Geelong, Victoria, 3216, Australia

[2]Telethon Kids Institute, University of Western Australia, Perth, WA 6009, Australia

[3]The College of Nursing and Health, Zhengzhou University, Zhengzhou, 450001, China

[4]Institute for Immunology and Infectious Diseases, Murdoch University, Perth, WA 6150, Australia

[5]Cancer Care Centre, St George Hospital, Kogarah, and St George and Sutherland Clinical School University of New South Wales Kensington, NSW 2217, Australia

[6]Institute for Frontier Materials, Deakin University, Waurn Ponds, Victoria 3216, Australia

[7]Laboratory of Tumor Molecular and Cellular Biology College of Life Sciences Shaanxi Normal University 620 West Chang'an Avenue, Xi'an, Shaanxi 710119, China

[8]CAS Key Laboratory of Nano-Bio Interface, Suzhou Institute of Nano-Tech and Nano- Bionics, Chinese Academy of Sciences, Suzhou 215123, China

[9]Shanghai OneTar Biomedicine, Shanghai, 201203, P. R. China

[10]College of Materials and Chemical Engineering, Minjiang University, Fuzhou, Fujian, 350108, China

[11]State Key Laboratory of Oncogenes and Related Genes, Shanghai, 200127, P. R. China

[12]Department of Biliary-Pancreatic Surgery, Renji Hospital Affiliated to Shanghai Jiao Tong University School of Medicine, Shanghai, 200127, P. R. China

[13]Shanghai Key Laboratory of Biliary Tract Disease Research, Shanghai, 200092, P. R. China

#These authors contributed equally to this work

ΔCurrent address: Nutromics Pty Ltd, 420 Victoria Street, Brunswick, Victoria 3056, Australia 3056

Supplementary Materials

***Corresponding authors:** Dongxi Xiang, Email: dxiang@shsmu.edu.cn; and Wei Duan, Email: wei.duan@deakin.edu.au.

Supplementary materials and methods

Cell lines and cell culture

PLC/PRF/5 (human hepatocellular carcinoma, ATCC CRL-8024) cell line, HEK-293T (human embryonic kidney, ATCC CRL-11268) cell line were purchased from American Type Culture Collection (ATCC, Manassas, US). Huh7 (human hepatocellular carcinoma, Japanese Collection of Research Bioresources) cell line was kindly provided by Dr. Liang Qiao (University of Sydney, Australia). All the above cells were cultured in DMEM (Invitrogen, Australia) medium supplemented with 10% fetal bovine serum (FBS, Bovogen, Australia), penicillin (50 U/mL, Invitrogen, Australia), streptomycin (50 µg/mL, Invitrogen, Australia) and 1 × Glutamax (Life Technologies) in a humidified atmosphere containing 5% CO_2 at 37 °C.

For sphere culture and passage, the adherent cells were collected and washed with PBS to remove serum, and then suspended in the stem cell culture medium including serum-free DMEM/F12 supplemented with 20 ng/ml human epidermal growth factor (EGF, R&D Systems, #701-02360), 20 ng/ml human basic fibroblast growth factor (bFGF, R&D Systems, #701- 23300), 5 µg/ml insulin (Sigma, #I9278) and 2% B27 supplement (Gibco, #10889-038). The cells were plated into a 6-well flat bottom ultra-low attachment plate (Corning, #CLS3474) at a density of 20,000 cells/well. After seven to ten days, the formation of tumorspheres was analyzed using an inverted microscope. Only spheres with a size larger than 50 µm in diameter and a well-defined border were counted as tumorspheres. The tumorspheres were collected and dissociated with trypsin to generate single cells and resuspended in a serum-free medium and allowed to re-form spheres. The tumorspheres were passaged every 7-8 days. The sphere-forming cells from Huh7 and PLC/PRF/5 were used in the subsequent studies.

Development of CD133 aptamer-DOX conjugates

CD133 aptamers were designed for conjugation with the chemotherapy agent DOX (Sigma, #44583), which was prepared at concentrations of 100 µM and stored at 4 °C. DOX was mixed well with folded aptamers in a conjugation buffer containing 0.1 M sodium acetate, 0.05 M NaCl, and 2.5 mM $MgCl_2$ and incubated in an orbital mixer/incubator (RATEK) at 75 r.p.m. for 1 h. The conjugate mixture was then passed through a Sephadex G-50 medium column (Sigma, #11814427001) to separate the conjugates from free DOX.

Determination of the molar ratio of aptamers to DOX

The secondary structure of CD133 aptamer was predicted by the Vienna online tool (http://rna.tbi.univie.ac.at/). DOX has fluorescence properties and its natural fluorescence becomes quenched after intercalation into DNA due to the formation of charge-transfer complexes between the ring structure of anthracyclines in DOX and the DNA [1]. This property is utilized to study how many DOX molecules can be intercalated into one CD133 aptamer molecule. First, the CD133 aptamers were folded in PBS with the presence of 2.5 mM Mg^{2+} by heating at 85 °C for 5 min, cooling to room

temperature for over 10 min, followed by incubation at 37 °C for 15 min. The folding procedure provides the aptamers with the correct three-dimensional structure. After folding, DOX was mixed well with different concentrations of aptamers in conjugation buffer and incubated at 37 °C in an orbital mixer/incubator (RATEK) at 75 r.p.m. for one hour. The conjugation process was studied using different aptamer-DOX molar ratios (0, 0.1, 0.2, 0.3, 0.4, 0.5, 0.6, 0.7, 0.8, 0.9 and 1) and DOX fluorescence was detected using a plate reader (470 nm excitation/585 nm emission) (Perkin Elmer) after intercalating with different concentrations of aptamers. The percentage of quenching is calculated as

$$\text{Quenching\%} = \frac{\text{DOX fluorescence in the presence of aptamer} - \text{background fluorescence}}{\text{DOX fluorescence in the absence of aptamer} - \text{background fluorescence}} \times 100\%$$

Determination of DOX loading efficiency

From the quenching assay, we determined the molar ratio of DOX to CD133 aptamers. To determine the loading efficiency, DOX was intercalated with the CD133 aptamer as described above with a ratio of 2:1. The prepared CD133 aptamer-DOX were eluted from a Sephadex G- 50 column to remove the free DOX. Thirty microliters of the eluate were mixed with 90 µL acetonitrile and vortexed for 1 min to extract DOX from the aptamer-DOX conjugates. This solution was centrifuged at 21,000 × *g* for 5 min, and 60 µL supernatant was added into the wells of the low-fluorescence plate. The fluorescence of the solution containing DOX was detected using a plate reader (485 nm excitation/585 nm emission). The concentration of DOX was determined using a standard curve generated by plotting fluorescence versus DOX with known concentration. The loading efficiency study was repeated three times with five replicates. DOX loading efficiency (DLE) was calculated as

$$\text{DLE} = \frac{\text{DOX loaded in the conjugates}}{\text{DOX added in the conjugates}} \times 100\%$$

Determination of the stability of CD133 aptamer-DOX

The stability of CD133 aptamer-DOX was studied by monitoring the release of DOX from CD133 aptamer-DOX using a Slide-A-Lyzer Dialysis Cassette (ThermoFisher). CD133 aptamer-DOX at an equivalent DOX concentration of 1 µg/mL was dialyzed against PBS at pH 5.0 or 7.4 at 37 °C. At various time points (0.5 h, 1 h, 2h, 4 h, 6 h, 8 h, 12 h, 24 h, 36 h, 48 h, and 72 h), 200 µL of each sample outside the dialysis cassette were collected and the concentration of free DOX in the dialysis buffer was measured.

Determination of CD133 aptamer-DOX binding affinity

Huh7, PLC/PRF/5 or HEK293T cells were first incubated with blocking buffer (PBS supplemented with 2.5 mM $MgCl_2$, 0.1 mg/mL tRNA, 0.1 mg/mL salmon sperm DNA, and 5% FBS) for 20 min at room temperature followed by two washes with PBS before incubation at serial concentrations (0, 10, 20, 40, 60, 80, 100, 150 and 200 nM) of Alexa 647-labeled CD133 aptamer and Alexa 647-labeled negative control aptamer respectively in a 100 µL blocking buffer at 37 °C for 30 min. The cells were

washed three times, resuspended in 150 μL assay buffer containing 2.5 mM $MgCl_2$, and subjected to flow cytometric analyses. The binding affinity was calculated after subtracting the mean fluorescence intensity (MFI) obtained from target cells from that of negative control cells according to a method described by Li and colleagues [2]. Fluorescent histograms were recorded by the BD FACS-Canto II flow cytometer and analyzed using the FlowJo V10 software.

Isothermal calorimetry (ITC)

ITC measurements were performed at 25 °C with a MicroCal PEAQ-ITC calorimeter (Malvern Panalytical, UK). CD133 aptamers were folded as described above. The sample cell contained 200 μl of aptamers (100 μM) in PBS with 2.5 mM $MgCl_2$, and the injection syringe contained 40 μl of DOX (100 μM) in the same solvent to minimize the heat of solvent dilution. The volume of each injection was 2 μl, except that the volume of the first injection was 0.4 μl. Nineteen injections were involved in each experiment at 150 s intervals between injections. The syringe rotational speed was 750 r.p.m. The reference power was 10 μcal/s. Dissociation constant Kd, the stoichiometry of binding N, and enthalpy change ΔH were calculated by the instrument software. The change of Gibbs free energy ΔH was calculated as $\Delta G = RT \times \text{In}K\text{d}$, where the universal gas constant $R = 8.314$ J mol^{-1} K^{-1}, T is the temperature on the Kelvin scale, and Kd is the dissociation constant obtained by the software. Entropy change ΔS was calculated as $\Delta S = (\Delta H - \Delta G)/T$.

siRNA transfection

The dicer-substrate siRNA against ATG5 was derived from a previously published 21-mer, which showed high knockdown efficiency [3] and was extended to a 27-mer dicer-substrate siRNA for enhanced silencing efficiency [4]. The scrambled siRNA has the same composition of nucleotide as the ATG5 siRNA but lacks significant sequence homology to the human genome. The scrambled siRNA was designed using the InvivoGen online tool (http://www.invivogen.com/sirnawizard/scrambled.php) and verified using the BLAST software tool (https://blast.ncbi.nlm.nih.gov/Blast.cgi). The siRNAs were synthesized by Integrated DNA Technologies (IDT) followed by HPLC purification. The siRNA sequences for ATG5 were 5'- GGCAUUAUCCAAUUGGUUUGCUAdTdT-3′ and 5'-AAUAGCAAACCAAUUGGAUAAUGCCAU-3′, and for scrambled siRNA sequences 5'- GGACUCUUCGGGAUUUACUAUUAdTdT-3′ and 5'-AAUAAUAGUAAAUCCCGAAGAGUCCAU-3′. Twenty-four h before siRNA transfection, cells were plated in a 6-well cell culture plate at a density of 3×10^5 cells/well in 1.5 mL/well DMEM medium without antibiotics. The ATG5 siRNA and their corresponding scrambled siRNAs at a concentration of 20 nM were gently diluted in 250 μL of Opti-MEM reduced serum medium, respectively. In parallel, 10 μL of Lipofectamine 2000 was diluted in 250 μL Opti-MEM medium. After incubation of 5 min at room temperature, the diluted siRNA and Lipofectamine 2000 were mixed gently and incubated for 10 min at room temperature, followed by the addition of 500 μL of siRNA-Lipofectamine complexes to each well (containing cells and 1.5 mL full DMEM medium).

The plate was mixed gently by rocking back and forth, and the cells were incubatedin a humidified atmosphere containing 5% CO2 at 37 °C for 72 h. Four hours before harvesting the cells, the lysosomal inhibitor chloroquine was added to the cell culture media to block the degradation of LC3-II. The expression levels of LC3-II were measured by Western analysis.

Protein extraction and western blot assays

Cells from adherent or non-adherent cultures were collected by trypsinization and transferred into a 1.5 or 15 mL tube. The cells were washed twice with PBS by centrifugation at 1,000 × *g* for 5 min, and the pellet was suspended in lysis buffer (20 mM Tris- HCl pH 7.5, 135 mM NaCl, 1.5 mM $MgCl_2$, 1 mM EDTA, 1% Triton X-100, 10% glycerol, and 1 × protease inhibitor cocktails (Roche) at a ratio of 0.2 mL/1 × 10^6 cells. Following 10 min of incubation on ice, the lysates were centrifuged at 21,500 × *g* at 4 °C for 30 min. The supernatant was then collected and used for Western blot analysis, with the remainder stored at -80 °C. Lysates were separated using SDS-polyacrylamide gel electrophoresis gels (PAGE), and the proteins were transferred onto a PVDF membrane (Thermo Scientific). Following extensive blocking with 5% skim milk (4 h at room temperature or overnight at 4 °C), primary antibodies were incubated according to the manufacturers' suggestions. Specific rabbit LC3-II (Cell Signaling, #2775) and mouse β-actin (Abcam, #ab6276) were detected using the goat anti-rabbit antibody (Cell Signaling, #7074) or the goat anti-mouse antibody (Thermo Fisher, #31430) respectively, and visualized using the Super Signal West Dura substrate (Thermo Fisher, #34076). Relative quantification was conducted using a LAS-4000 Imaging System (GE Healthcare Life Sciences) with β-actin as a loading control.

Inhibition of endocytosis

The cells were pre-treated with a potassium-depleted buffer (50 mM HEPES, 140 mM NaCl, 2.5 mM $MgCl_2$, and 1 mM $CaCl_2$) for 1 hour at 37 °C before incubation with the aptamers. The buffer was also used in the incubation step with aptamers and all rinsing steps. The effectiveness of these treatments in inhibiting endocytosis was evaluated by qualitatively characterizing the internalization of human transferrin conjugated to Alexa Fluor 488 (Life Technologies, #T13342). Transferrin (5 μg/mL) was added to the cells following pre-treatment and incubated for 30 min at 37 °C. The cells were washed three times in their respective buffers and visualized using a FluoView FV10i confocal microscope (Olympus).

Cellular uptake and retention of DOX

Huh7, PLC/PRF/5, and HEK293T cells were seeded at 8 × 10^3 cells per well in an 8-chamber slide (Lab-Tek® II, Nunc, US) for 24 h in preparation for confocal microscopy. After 24 h the medium in the chamber slide was removed and 200 μL blocking buffer (PBS with 10% FBS, 0.1 ml/ml yeast tRNA, and 0.1 mg/ml herring sperm DNA) was added into each well followed by 20 min incubation at room temperature. The blocking buffer was removed, and cells were washed twice with PBS. The

cells were incubated with DOX, CD133 aptamer-DOX, and negative CD133 aptamer-DOX for 40 min, respectively. The concentration of DOX used was 400 nM. Bisbenzimide Hoechst 33342 (3 μg/ml) (Sigma, #14533) was added to the cells during the final 10 min of incubation. Cells were washed with PBS three times before imaging.

Chromatographic instrumentation and system

To quantitatively determine the accumulation of DOX inside cancer cells, cellular uptake and retention of free DOX were determined using HPLC. After trypsinization, the cells were washed with PBS and lysed with 200 μL of cell lysate buffer (50 mM Tris pH 7.5, 375 mM NaCl, 1 mM EDTA pH 8.0, 8% glycerol, and protein inhibitor cocktail). Four hundred microliter of acetonitrile was added to each sample precipitate protein. The samples were vortexed for 30 seconds and centrifuged at 20,000 × *g* for 10 min. DOX concentration was quantified using HPLC consisting of a Waters e2695 separation module and a Waters 2475 Multi λ fluorescence detector. The excitation and emission wavelengths were set at 470 nm and 585 nm, respectively. Chromatographic separation was performed using a Nova-Pak C18 column (3.9 × 150 mm i.d., 4 μm, Waters, US) with a Nova-Pak C18 guard column (3.9 × 20 mm i.d., 4 μm, Waters, US). A mixture (27:73, v/v) of acetonitrile and 10 mM phosphate buffer (pH = 3.0, Fluka) was used as the mobile phase. The flow rate used in the assay was 1 mL/ minute, and the column was maintained at 35 ± 5 °C throughout the chromatographic process. All solvents for HPLC procedures were prepared freshly and filtered with a 0.22 μm membrane before use.

Cell surface marker analysis

For cell surface marker analysis, cells from cell culture were dissociated into single cells, washed with PBS containing 0.1% BSA, and stained with human-specific antibodies to APC/Fire 750- conjugated EpCAM (Biolegend, #324234) and VioBright FITC-conjugated CD133 (Miltenyl Biotec, #130-105-225) for 20 min at 4 °C. After thorough washing with PBS, the cells were stained with 7-AAD for 5 min, and the population of liver cancer stem cells (defined as $EpCAM^+$ - $CD133^+$) was analyzed using flow cytometry. Isotype and conjugate-matched antibodies were used as negative controls, including the APC/Fire 750 mouse IgG2b, κ isotype control (Biolegend, #400372), and Mouse IgG1-VioBright FITC isotype control (Miltenyl Biotec, #130-104-513). A minimum of 10,000 events was analyzed for each sample from three independent experiments.

Tunnel assay

The TUNEL assay was used to determine late-stage cell apoptosis [5]. The apoptotic responses were determined using Click-iT® TUNEL Alexa Fluor® 488 imaging assay kit according to the manufacturer's protocol (Thermo Fisher, #C10245). In brief, cells were fixed with 4% paraformaldehyde for 10 min at room temperature. One hundred microliters of cell suspension were moved to a microscope slide, followed by washing with 2 changes of PBS. The cells were then incubated with permeabilization reagent Triton X-100 (0.25%) for 20 min at room temperature. The terminal

deoxynucleotidyl transferase (TdT) reaction cocktail was applied to the coverslips, and the cells were incubated for 60 min at 37 °C in a humidified chamber. After being washed twice with PBS containing 3% BSA, the coverslips were incubated with a reaction cocktail for 30 min at room temperature and prevented from light. The reaction cocktail was removed, and the coverslips were washed with PBS containing 3% BSA. The coverslips were incubated with Hoechst 343342 (2 µg/mL) to stain cellular DNA for 15 min at room temperature, followed by washing with PBS twice. The cells were visualized with the Fluoview FV10i laser scanning confocal microscope (Olympus, NSW, Australia).

In vitro tumorsphere formation assay

The tumorsphere assay was conducted according to our previously published protocol [6] with modifications. The details can be found in the supplementary materials. Briefly, cells were seeded in 6-well cell culture plates and treated with the following reagents for 48 h: PBS, salinomycin, DOX, CD133 aptamer-DOX, 3-methyladenine (3-MA), DOX plus 3-MA, and CD133 aptamer-DOX plus 3-MA respectively. The concentration of the positive control treatment salinomycin was 2 µM to Huh7 cells and 0.6 µM to PLC/PRF/5 cells; DOX and CD133 aptamer- DOX concentrations were 200 nM to Huh7 cells and 100 nM to PLC/PRF/5 cells; 3-MA concentration was 2 mM. Cells were harvested at 80% confluence with trypsin digestion and resuspended as single cells in the stem cell culture medium in 96-well ultra-low attachment plates at a density of 10, 20, and 50 cells per well, respectively at 37 °C. The reagents with the same concentration were added to the sphere culture medium for the rest of the whole culture period. After 7 days, the formation of tumorspheres was visualized by light microscopy. The frequency of cancer stem cells was calculated using the Extreme Limiting Dilution Analysis online tool (http://bioinf.wehi.edu.au/software/elda/index.html).

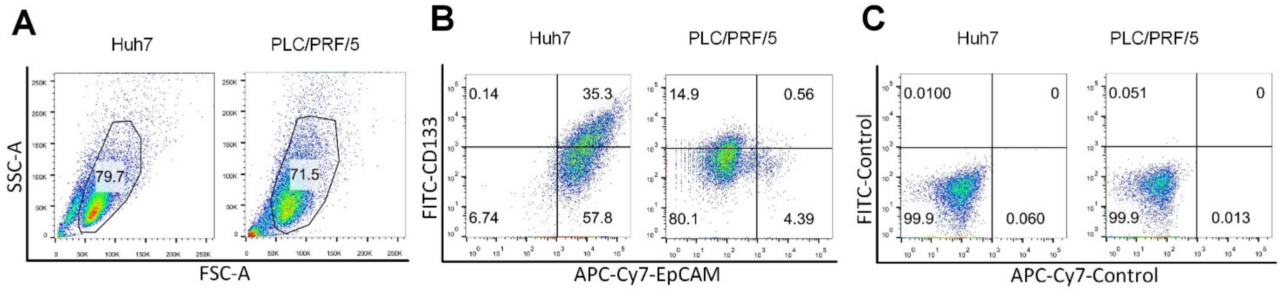

Supplementary Figure S1. The expression of cancer stem cell markers EpCAM and CD133 on Huh7 and PLC/PRF/5 cells. (A) The gating of viable and single cells using forward scatter (FSC) and side scatter (SSC). **(B)** The percentage of EpCAM$^+$ and CD133$^+$ population in the Huh7 and PLC/PRF/5 cells. **(C)** The Huh7 and PLC/PRF/5 cells were stained with IgG isotope-matched control antibodies for EpCAM and CD133. The horizontal axis denotes fluorescence intensity, and the vertical axis denotes cell counts **(A)** or fluorescence intensity **(B** and **C)**. Data shown are representative of three independent experiments.

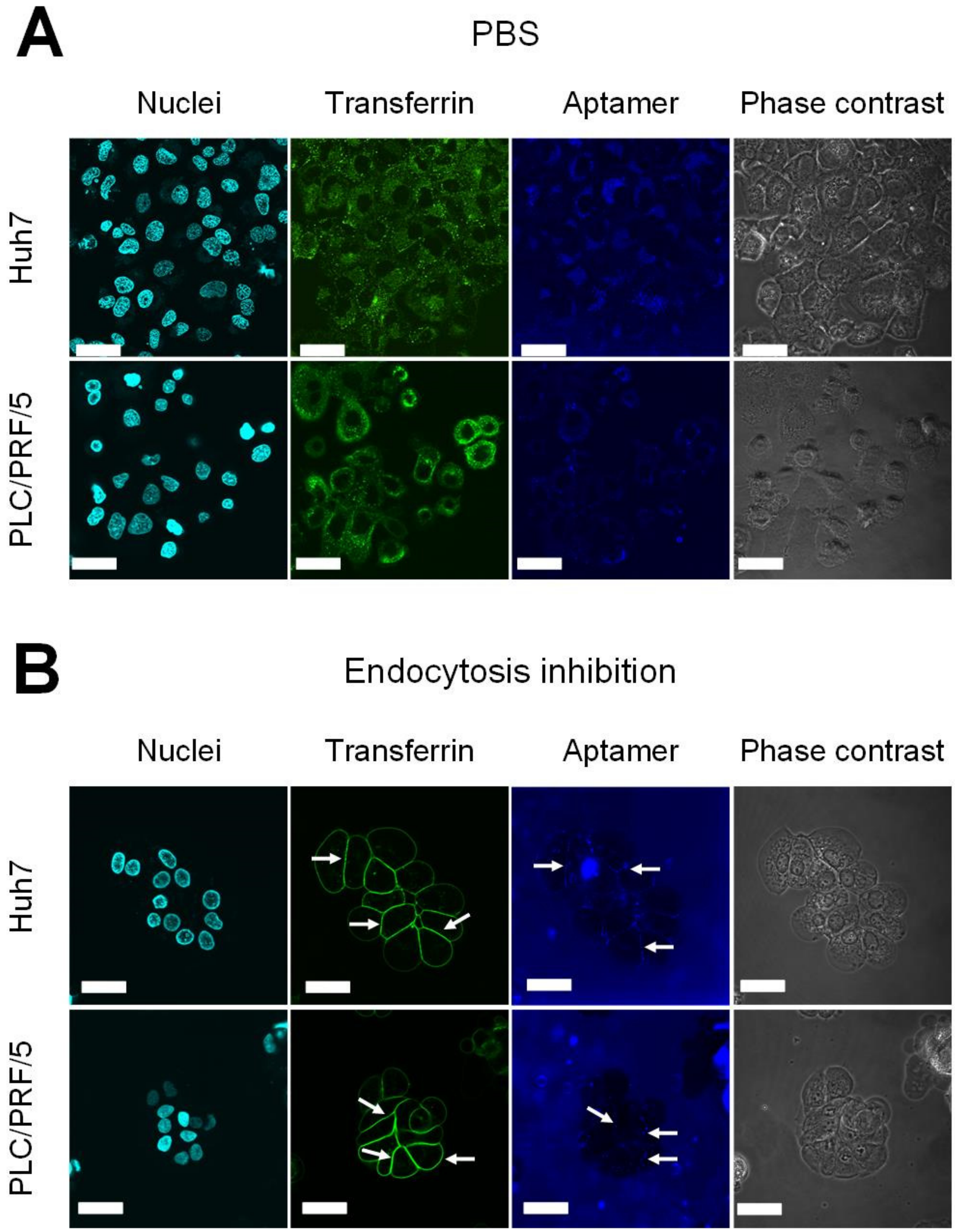

Supplementary Figure S2. CD133 aptamer is endocytosed via receptor-mediated endocytosis. **(A)** Representative micrographs showed Huh7 or PLC/PRF/5 cells incubated with Alexa 647-labeled CD133 aptamers for 60 min at 37 °C, followed by imaging using laser scanning confocal microscopy. **(B)** Huh7 and PLC/PRF/5 cells were treated with potassium-depleted buffer to inhibit endocytosis, followed by the incubation with transferrin and Alexa 647-labeled CD133 aptamer for 60 min at 37 °C, and subjected to imaging using laser scanning confocal microscopy. Cyan: nuclear stain; green: transferrin; blue: CD133 aptamer stain. Scale bar = 40 µm.

1

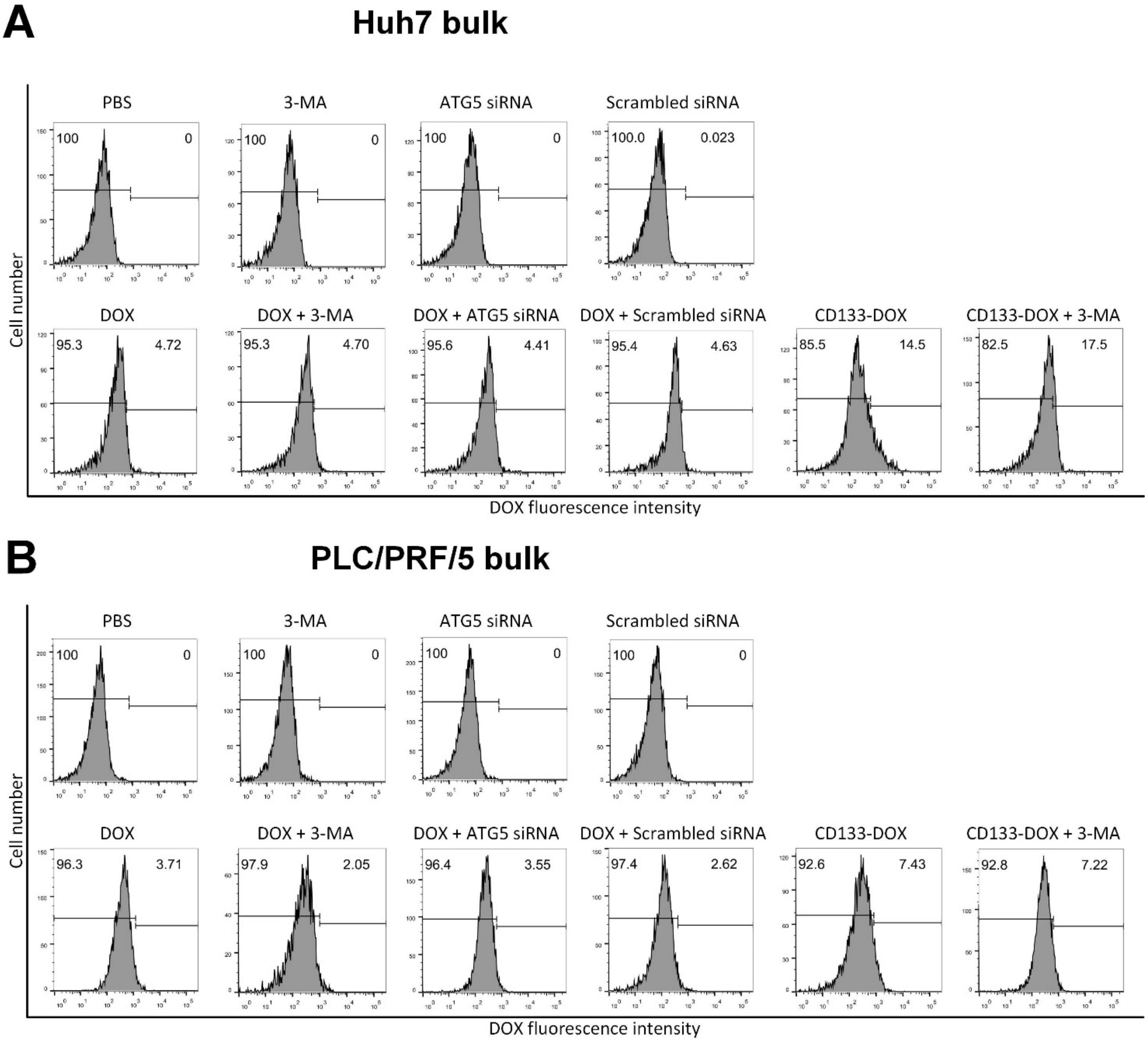

Supplementary Figure S3. Representative flow cytometric diagrams showing DOX accumulation in the bulk population of (A) Huh7 or (B) PLC/PRF/5 cells after treatment with DOX (200 nM for Huh7 and 100 nM for PLC/PRF/5), 3-MA (2 mM), ATG5 knockdown (20 nM siRNA), CD133 aptamer-DOX (200 nM or 100 nM equivalent DOX for Huh7 or PLC/PRF/5), indicated combined treatment or vehicle control treatment for 24 h. One representative experiment of three is shown.

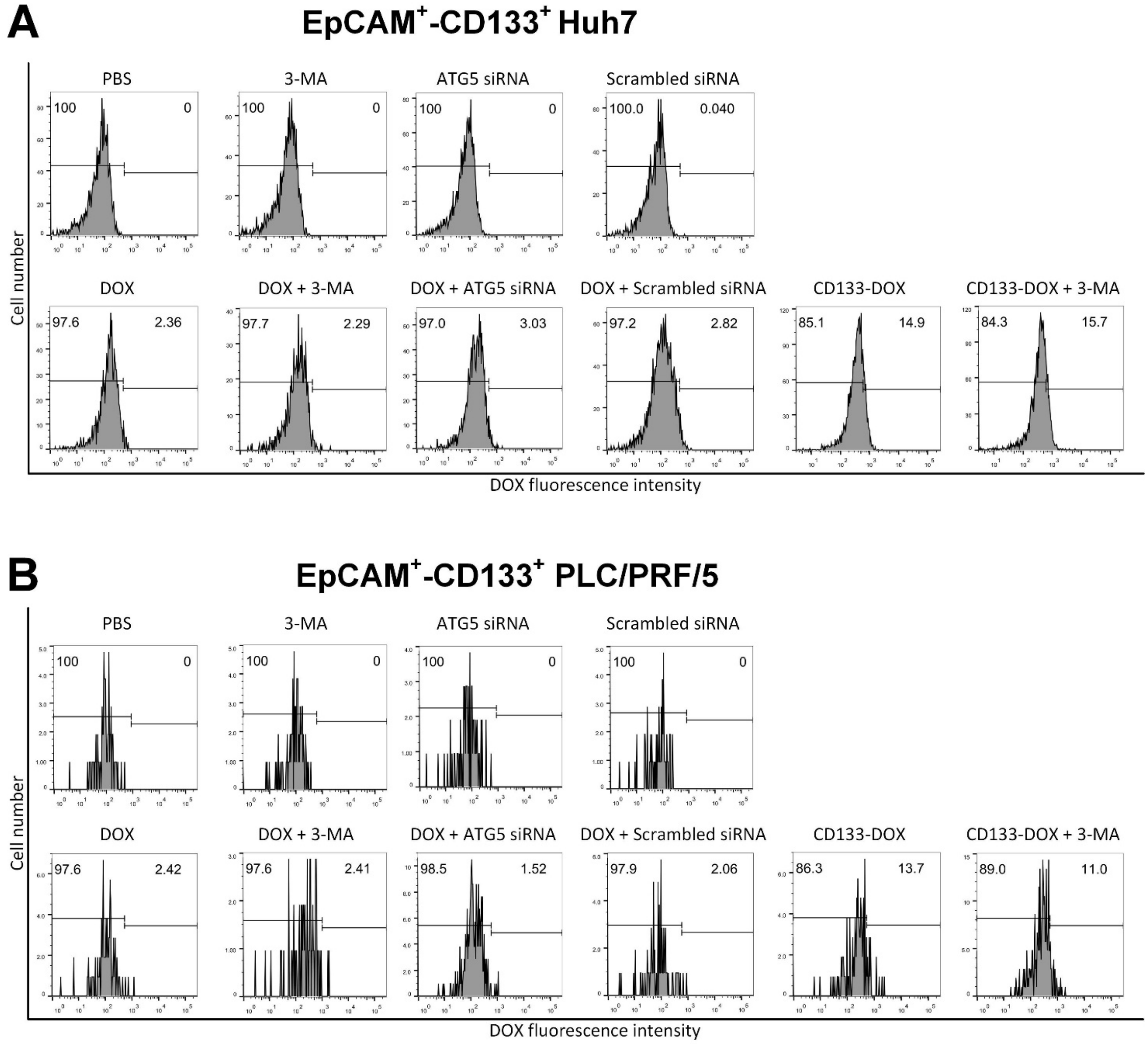

Supplementary Figure S4. Representative flow cytometric diagrams showing DOX accumulation in the EpCAM$^+$-CD133$^+$ population of (A) Huh7 or (B) PLC/PRF/5 cells after treatment with DOX (200 nM for Huh7 and 100 nM for PLC/PRF/5), 3-MA (2 mM), ATG5 knockdown (20 nM siRNA), CD133 aptamer-DOX (200 nM or 100 nM equivalent DOX for Huh7 or PLC/PRF/5), indicated combined treatment or vehicle control treatment for 24 h. One representative experiment of three is shown.

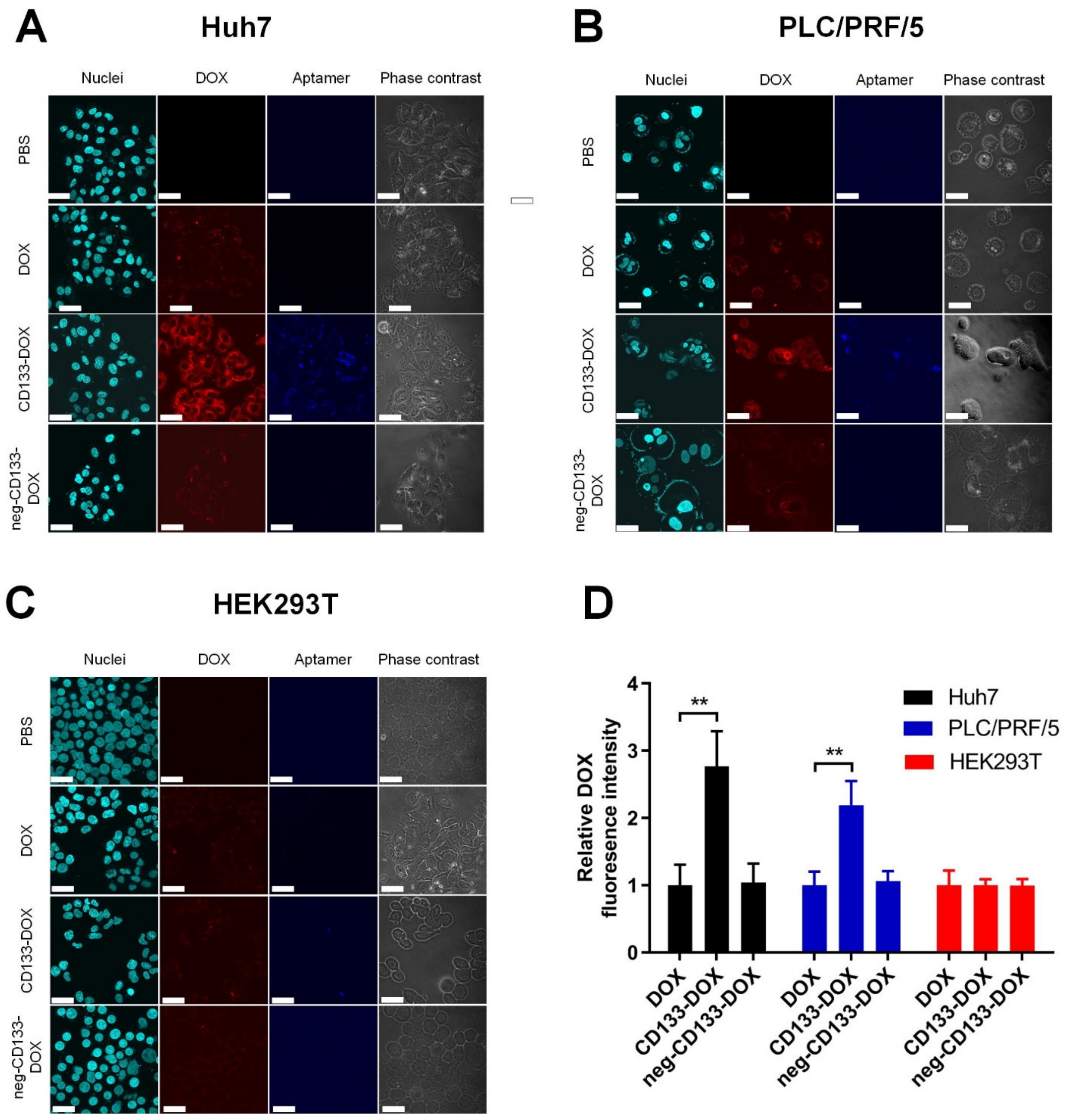

Supplementary Figure S5. Cellular uptake of DOX and CD133 aptamer-DOX in (A) Huh7, (B) PLC/PRF/5, and (C) HEK293T cells. The Huh7, PLC/PRF/5, or HEK293T cells were incubated with DOX, CD133 aptamer-DOX, or negative control CD133 aptamer-doxorubicin (neg-CD133 aptamer-DOX) with an equivalent DOX concentration of 400 nM for 40 min. **(D)** The uptake of CD133 aptamer-DOX in Huh7 and PLC/PRF/5 cells was quantified using the intracellular fluorescence of DOX using the ImageJ software (NIH, US). Scale bar = 40 µm. ****$p<0.0001$, compared to DOX treatment. Data shown are means ± SD, (n = 3)**.**

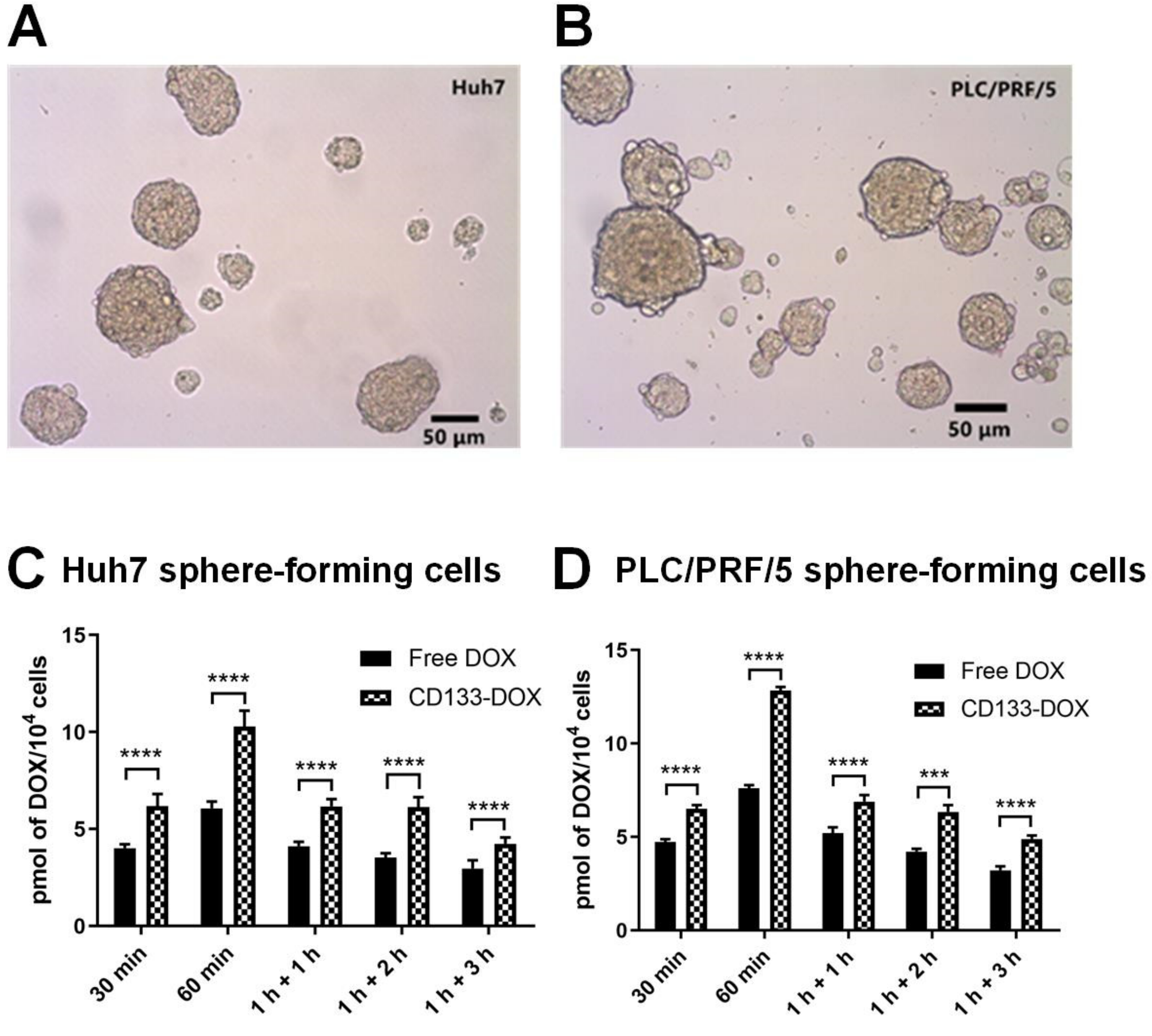

Supplementary Figure S6. In vitro tumorsphere assay. The liver cancer cells were grown in serum-free, non-adherent conditions for the enrichment of cancer stem cells, as only the cancer stem cells can survive and proliferate in this environment by self-renewal while differentiated cells undergo necrosis. Representative images for tumorsphere, defined as a solid and round structure with a clear border and a diameter of over 50 µm, were shown for **(A)** Huh7 and **(B)** PLC/PRF/5 cells. The cellular uptake and retention of CD133 aptamer-DOX in the sphere-forming cells derived are shown from **(C)** Huh7 cells and **(D)** PLC/PRF/5 cells. To investigate cellular uptake, the cells were incubated with either free DOX (100 nM) or CD133 aptamer-DOX (100 nM equivalent DOX) for 30 and 60 min, respectively. To investigate cellular retention, the sphere-forming cells were incubated with the same concentration of free DOX or CD133 aptamer-DOX for 1 h, washed with PBS three times, followed by further incubation for 1, 2, or 3 h in the drug-free cell culture medium followed by the quantification of fluorescence from intracellular DOX. Data shown are means ± SD, (n = 3). ***p<0.001; ****p<0.0001; compared to free DOX treatment.

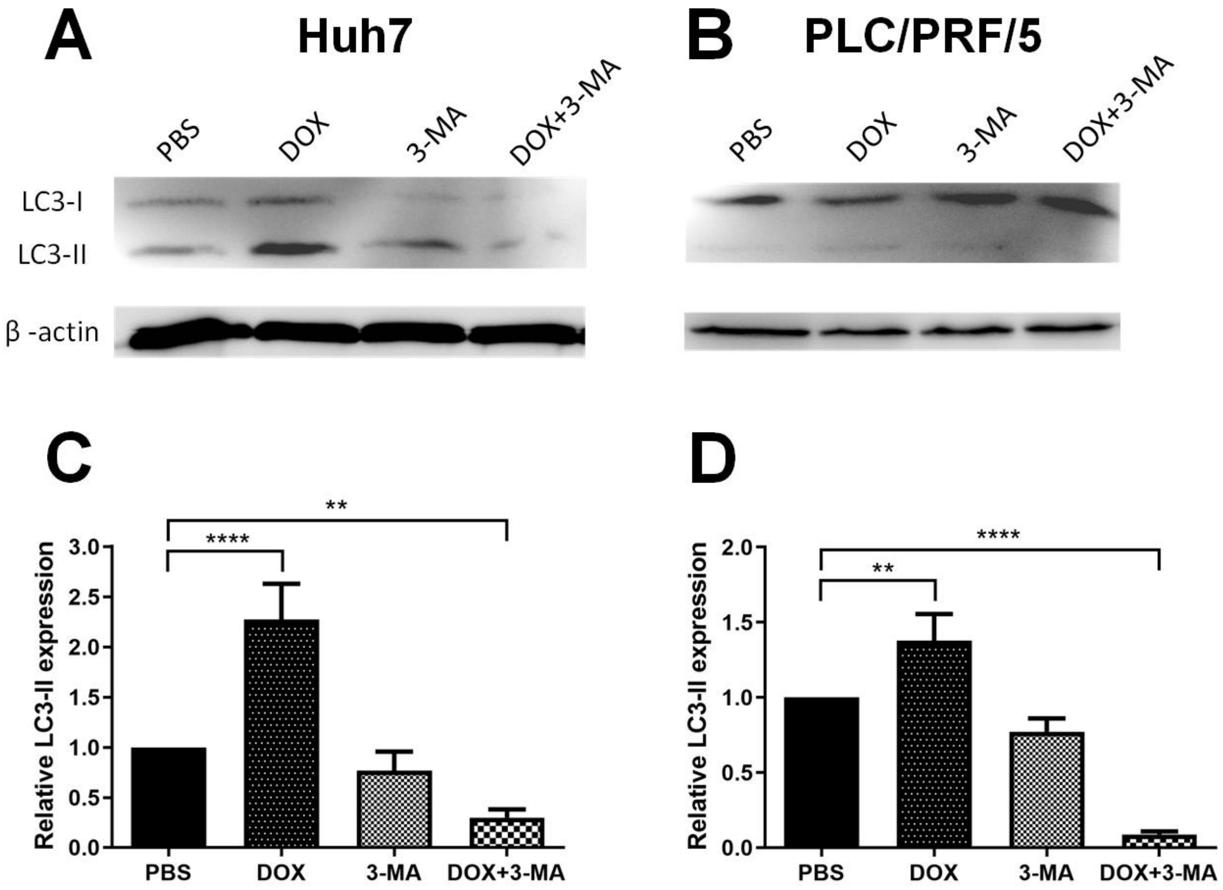

Supplementary Figure S7. Evaluation of the impact of DOX and 3-MA on autophagy. The Huh7 and PLC/PRF/5 cells were treated with PBS, DOX (200 nM for Huh7 and 100 nM for PLC/PRF/5), 3-MA (2 mM), and combined DOX and 3-MA for 2 days. Four hours before harvesting the cells, the lysosomal inhibitor chloroquine (40 µM) was added to the cell culture media to block the degradation of LC3-II. The cells were lysed, and the amount of LC3-II was determined by Western blotting analysis. **(A)** and **(B)**, The representative Western blotting images of LC3-I, LC3-II, and β-actin from **(A)** Huh7 or **(B)** PLC/PRF/5 cell lysate, respectively. **(C)** and **(D)**, Quantification of LC3- II in **(C)** Huh7 and **(D)** PLC/PRF/5 cells, respectively. LC3-II levels were normalized with that of β-actin. Data shown are means ± SD, (n = 3). ***p< 0.01; ****p< 0.001; *****p< 0.0001 compared to PBS treatment.

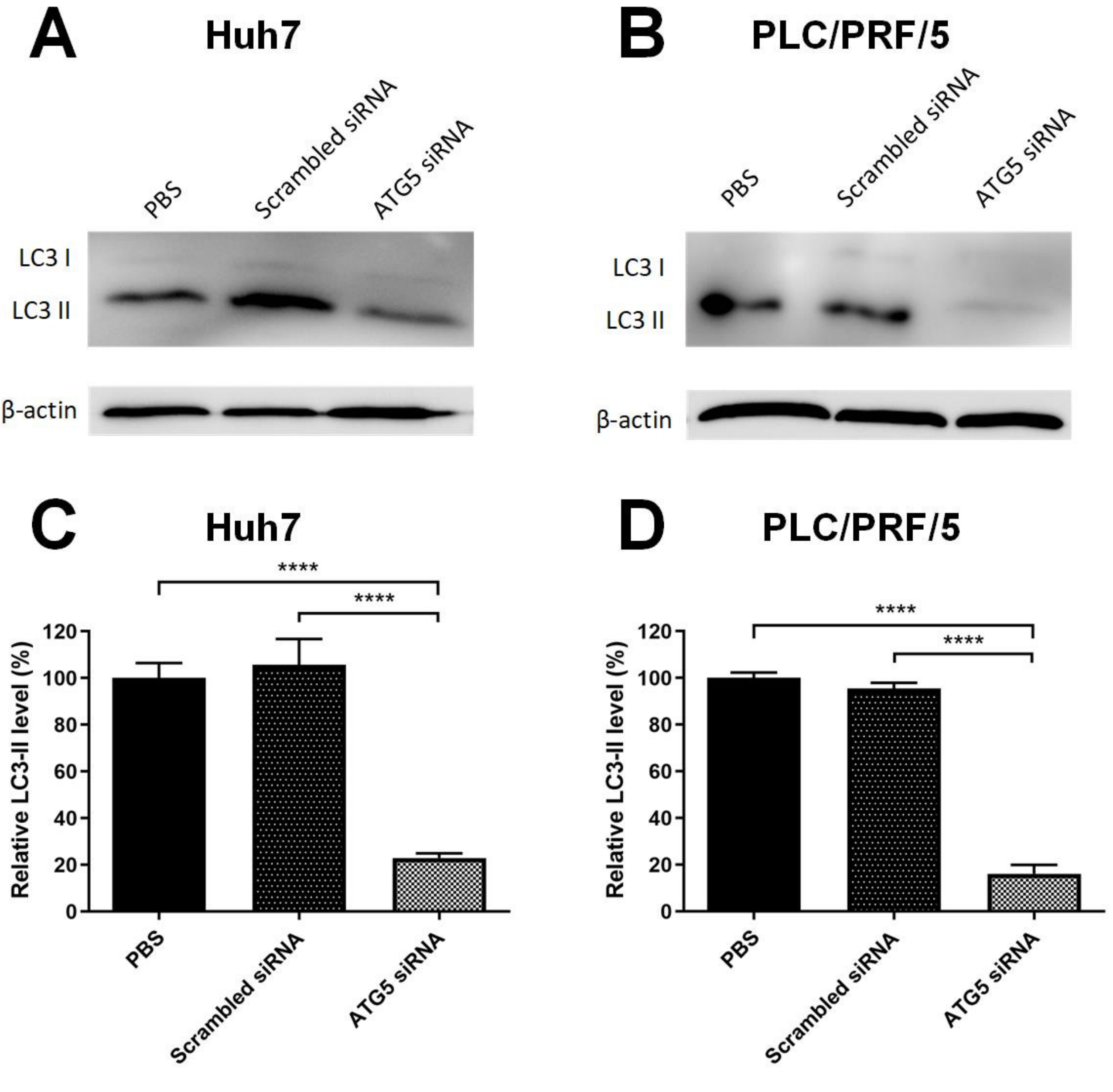

Supplementary Figure S8. ATG5 knockdown induced inhibition of autophagic activity. Representative Western blotting images of LC3-II expression after the downregulation of ATG5 via RNAi in **(A)** Huh7 and **(B)** PLC/PRF/5 cells. Treatment with 20 nM ATG5 siRNA resulted in >75% reduction of the LC3-II protein, which is widely used as a surrogate for autophagic activity in **(C)** Huh7 and **(D)** PLC/PRF/5 cells. Data shown are means ± SD, (n = 3). ***p<0.001; ****p<0.0001 compared to PBS or scrambled control siRNA group.

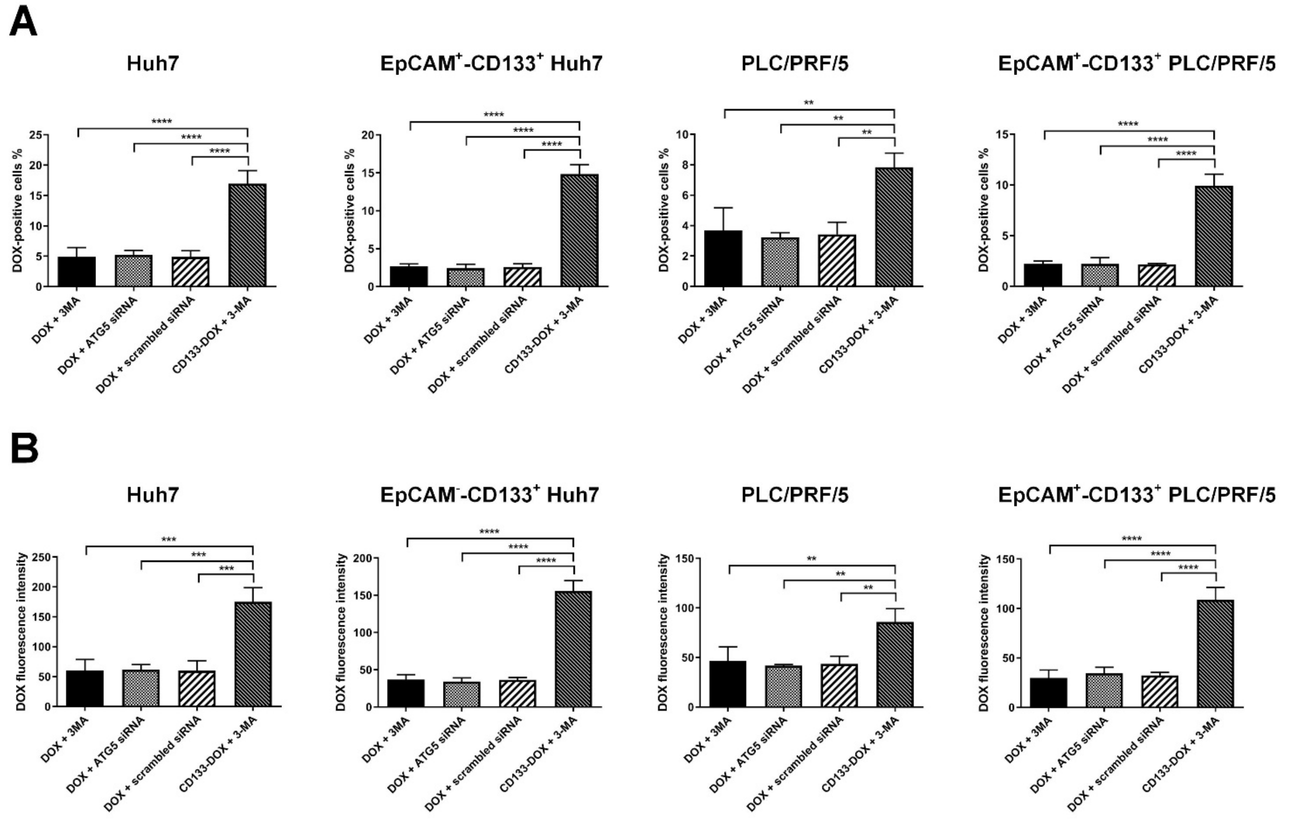

Supplementary Figure S9. Flow cytometric measurement of DOX accumulation in the bulk and EpCAM$^+$-CD133$^+$ population of Huh7 and PLC/PRF/5 cells followed by treatment of DOX plus 3-MA, DOX plus ATG5 siRNA knockdown, DOX plus scrambled control siRNA or CD133 aptamer-DOX plus 3-MA for 24 h. DOX and CD133 aptamer-DOX concentrations used were 200 nM for Huh7 and 100 nM for PLC/PRF/5. Furthermore, 2 mM 3-MA and 20 nM siRNAs were used. **(A)** The percentage of DOX-positive cells and **(B)** the corresponding DOX fluorescence intensity were presented. Data shown are mean ± SD, n = 3. **$p < 0.01$; ***$p < 0.001$; ****$p < 0.0001$ compared to CD133 aptamer-DOX plus 3-MA treatment.

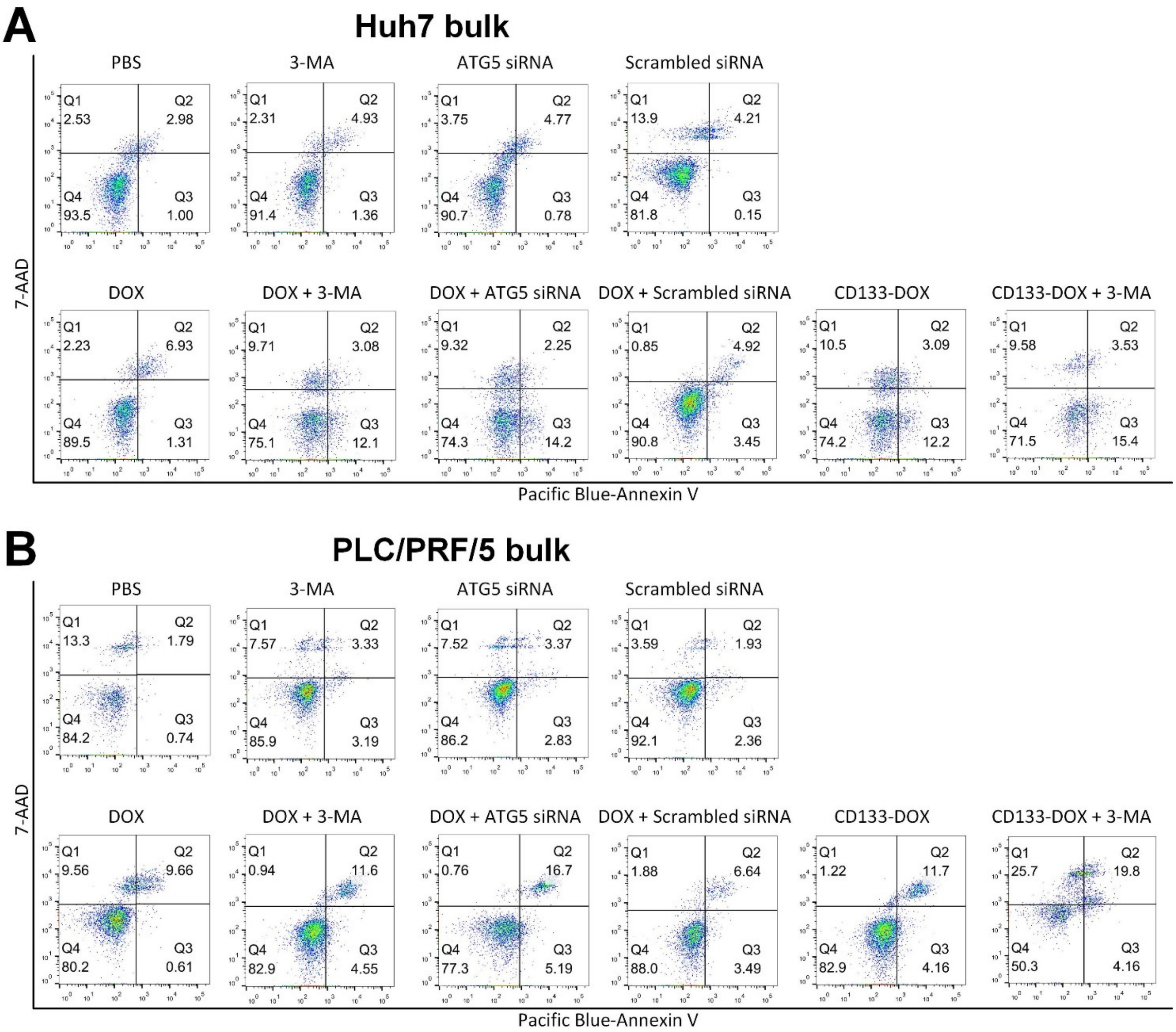

Supplementary Figure S10. **The contour diagram of 7-AAD/Annexin V flow cytometry.** The fluorescence profiles are shown for **(A)** Huh7 cells and **(B)** PLC/PRF/5 cells after treatment with DOX (200 nM for Huh7 and 100 nM for PLC/PRF/5), 3-MA (2 mM), ATG5 knockdown (20 nM siRNA), CD133 aptamer-DOX (200 nM or 100 nM equivalent DOX for Huh7 or PLC/PRF/5), combinational treatment or negative controls for 24 h. In each composite panel, the lower left quadrant indicates viable cells (7-AAD$^-$/Annexin V$^-$); the lower right quadrant indicates early apoptotic cells (7-AAD$^-$/Annexin V$^+$); while the upper right quadrant contains late apoptotic cells (7-AAD$^+$/Annexin V$^+$). The combination of early (7-AAD$^-$/Annexin V$^+$) and late (7-AAD$^+$/Annexin V$^+$) apoptotic cells were used to define the apoptotic cells.

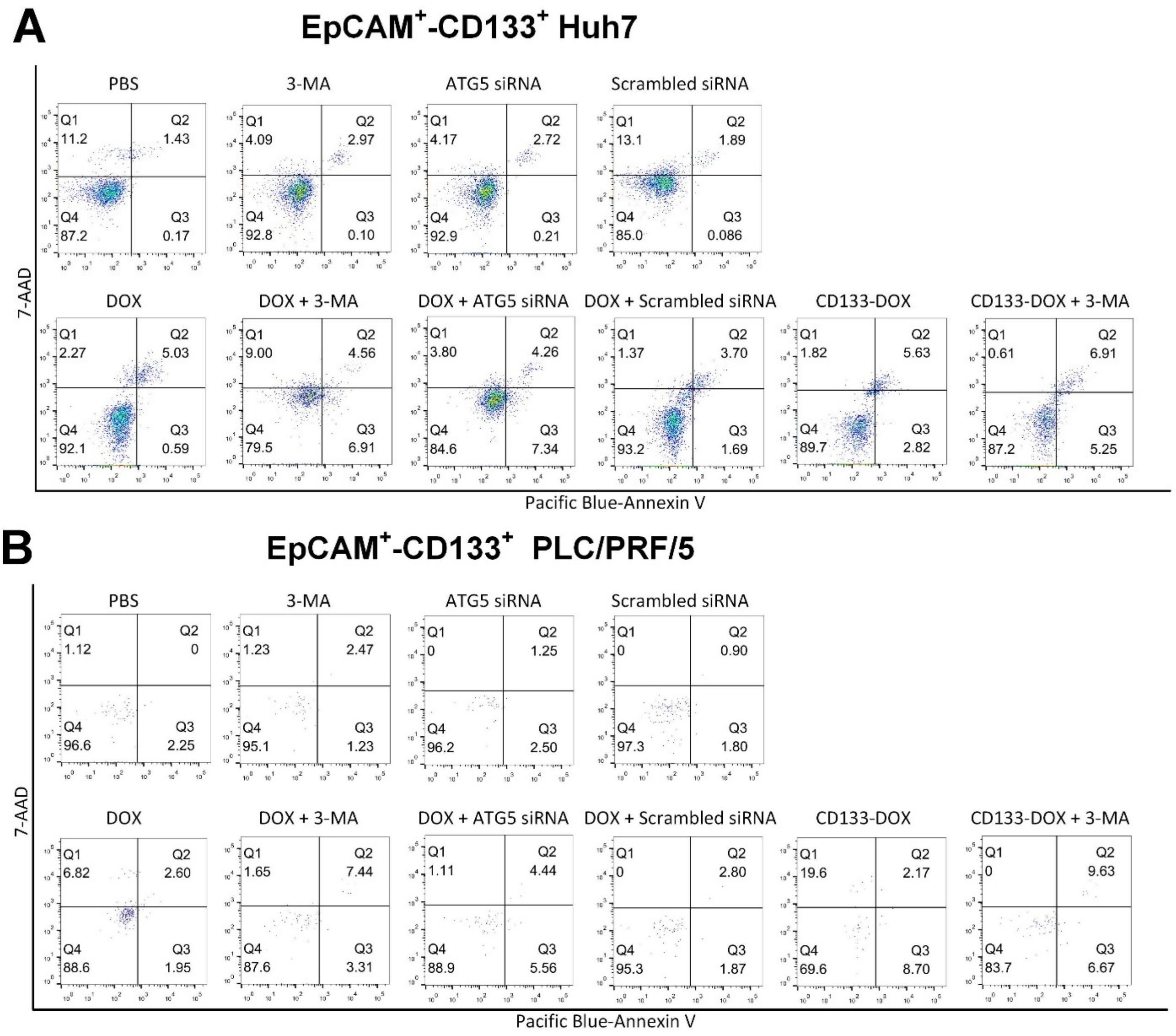

Supplementary Figure S11. The contour diagram of 7-AAD/Annexin V flow cytometry of the EpCAM$^+$-CD133$^+$ population. The representative fluorescence profiles are shown for **(A)** Huh7 cells and **(B)** PLC/PRF/5 cells after treatment with DOX (200 nM for Huh7 and 100 nM for PLC/PRF/5), 3-MA (2 mM), ATG5 knockdown (20 nM siRNA), CD133 aptamer-DOX (200 nM or 100 nM equivalent DOX for Huh7 or PLC/PRF/5), combined treatment or the corresponding negative controls for 24 h. In each composite panel, the lower left quadrant indicates viable cells (7-AAD$^-$/Annexin V$^-$); the lower right quadrant indicates early apoptotic cells (7-AAD$^-$/Annexin V$^+$); while the upper right quadrant contains late apoptotic cells (7-AAD$^+$/Annexin V$^+$). The combination of early (7-AAD$^-$/Annexin V$^+$) and late (7-AAD$^+$/Annexin V$^+$) apoptotic cells were used to define the apoptotic cells.

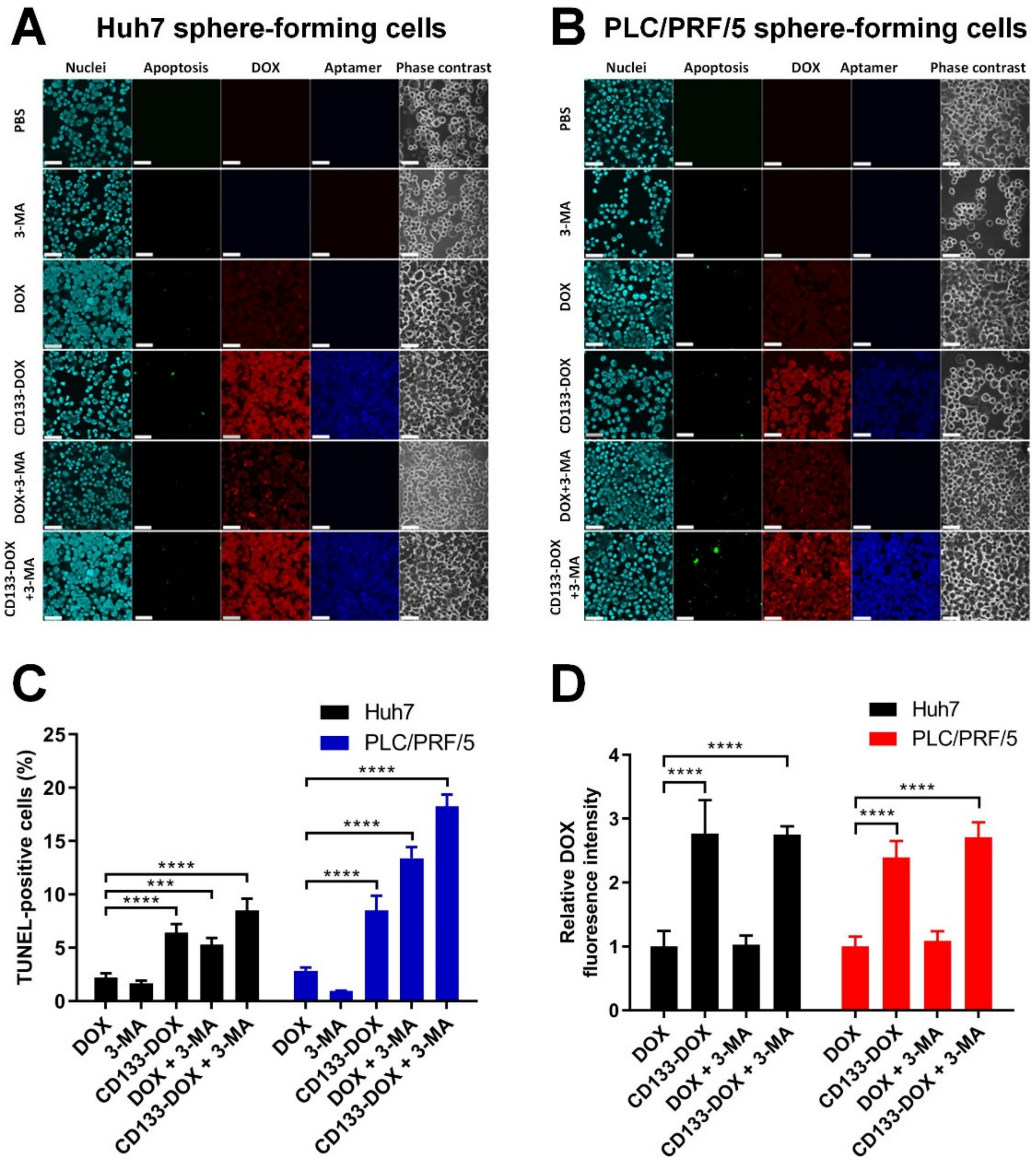

Supplementary Figure S12. Induction of apoptosis in the sphere-forming liver cancer cells. The apoptosis is shown following treatment with CD133-targeted delivery of DOX or autophagy inhibition combined with DOX treatment or 3-MA plus DOX treatments. Representative images of TUNEL apoptosis assay on the sphere-forming cells are presented for **(A)** Huh7 or **(B)** PLC/PRF/5 cells 48 h after the treatment by the agents as indicated on the left. Cyan: nuclear stain; green: apoptosis; red: DOX stain; blue: aptamer stain. Scale bar = 40 µm. **(C)** Percentage of apoptotic cells in treated sphere-forming cancer cells as determined by TUNEL assay. **(D)** Relative fluorescence intensity of DOX in cells represented as the fold to that treated with DOX alone. Data shown are mean ± SD, n = 3. ***p<0.001; ****p<0.0001, compared to free DOX treatment.

Supplementary Table S1. Effect of autophagy inhibition on the capability of DOX or CD133 aptamer-DOX to eliminate cancer stem cells in vitro*.

A) Huh7

Treatment	No. of cells per well	Tumorsphere incidence (out of six)
PBS	50	6.00±0.00
	20	6.00±0.00
	10	6.00±0.00
Salinomycin	50	1.00±0.00
	20	0.67±0.58
	10	0.00±0.00
DOX	50	6.00±0.00
	20	5.33±0.58
	10	5.00±1.00
3-MA	50	6.00±0.00
	20	4.33±0.58
	10	5.00±0.00
DOX + 3-MA	50	3.00±1.00
	20	1.67±0.58
	10	1.00±1.00
CD133-DOX	50	4.00 ±1.00
	20	1.67±0.58
	10	0.67±0.58
CD133-DOX + 3-MA	50	2.00±0.00
	20	0.33±0.58
	10	0.33±0.58

B) PLC/PRF/5

Treatment	No. of cells per well	Tumorsphere incidence (out of six)
PBS	50	6.00±0.00
	20	6.00±0.00
	10	6.00±0.00
Salinomycin	50	0.67±0.58
	20	0.67±0.58
	10	0.00±0.00
DOX	50	6.00±0.00
	20	5.33±0.58
	10	5.33±0.58
3-MA	50	6.00±0.00
	20	5.67±0.58
	10	4.00±1.73
DOX + 3-MA	50	3.33±0.58
	20	1.33±0.58
	10	1.00±1.00
CD133-DOX	50	3.33 ±1.15
	20	1.00±0.00
	10	0.33±0.58
CD133-DOX + 3-MA	50	1.67±0.58
	20	0.67±0.58
	10	0.00±0.00

*The cells were treated as described in the legend of Fig. 5. Data shown are mean ± SD.

References

[1] HT Haj, M. Salerno, W. Priebe, H. Kozlowski, A. Garnier-Suillerot, New findings in the study on the intercalation of bisdaunorubicin and its monomeric analogues with naked and nucleus DNA, Chemico-biological interactions, 145 (2003) 349-358.

[2] N. Li, J.N. Ebright, G.M. Stovall, X. Chen, H.H. Nguyen, A. Singh, A. Syrett, A.D. Ellington, Technical and biological issues relevant to cell typing with aptamers, J Proteome Res, 8 (2009) 2438-2448.

[3] J.M. Park, D. Tougeron, S. Huang, K. Okamoto, F.A. Sinicrope, Beclin 1 and UVRAG confer protection from radiation-induced DNA damage and maintain centrosome stability in colorectal cancer cells, PLoS One, 9 (2014) e100819.

[4] D.H. Kim, M.A. Behlke, S.D. Rose, M.S. Chang, S. Choi, J.J. Rossi, Synthetic dsRNA Dicer substrates enhance RNAi potency and efficacy, Nat Biotechnol, 23 (2005) 222-226.

[5] V.M. Heatwole, TUNEL assay for apoptotic cells, Methods in molecular biology (Clifton, N.J.), 115 (1999) 141-148.

[6] D. Xiang, S. Shigdar, A.G. Bean, M. Bruce, W. Yang, M. Mathesh, T. Wang, W. Yin,P.H.-L. Tran, H. Al Shamaileh, R.A. Barrero, P.-Z. Zhang, Y. Li, L. Kong, K. Liu, S.-F. Zhou, Y. Hou, A. He, W. Duan, Transforming doxorubicin into a cancer stem cell killer via EpCAM aptamer- mediated delivery, Theranostics, 7 (2017) 4071-4086.
